# Yttrium Oxide-Deficient Melts Control Secondary-Phase Distribution in Cerium-Doped Terbium–Yttrium Aluminum Garnet Crystals for White-Light Conversion and X‑ray Imaging

**DOI:** 10.1021/acs.chemmater.6c01589

**Published:** 2026-08-26

**Authors:** Karol Bartosiewicz, Justyna Zeler, Marcin E. Witkowski, Masao Yoshino, Robert Tomala, Om Prakash, Damian Szymanski, Maksym Buryi, Aleksandra Owczarek, Andrey Prokhorov, Vítězslav Jarý, Kei Kamada, Pietro Galinetto, Winicjusz Drozdowski, Eugeniusz Zych, Akira Yoshikawa, Gongxun Bai

**Affiliations:** † Institute of Physics of the Czech Academy of Sciences, Na Slovance 1999/2, 182 00 Prague 8, Czechia; ‡ Faculty of Chemistry, 49572 University of Wrocław, Joliot-Curie 14F, 50-383 Wrocław, Poland; § Institute of Physics, Faculty of Physics, Astronomy and Informatics, 49577 Nicolaus Copernicus University in Toruń, Grudziadzka 5, 87-100 Toruń, Poland; ∥ New Industry Creation Hatchery Center, Tohoku University, 6-6-10 Aramaki Aza Aoba, Aoba-ku, Sendai, Miyagi 980-8579, Japan; ⊥ Institute of Low Temperature and Structure Research, Polish Academy of Sciences, Okólna 2, 50-422 Wrocław, Poland; # Department of Physics, University of Pavia, Via Bassi 6, 27100 Pavia, Italy; ∇ Institute of Plasma Physics of the Czech Academy of Sciences, U Slovanky 2525/1a, 182 00 Prague 8, Czechia; ○ Institute for Materials Research, Tohoku University, 2-1-1 Katahira, Aoba-ku, Sendai, Miyagi 980-8577, Japan; ◆ College of Science, 92270 China Jiliang University, Hangzhou 310018, China

## Abstract

Controlled melt nonstoichiometry was investigated as a processing parameter governing secondary-phase formation and functional properties of Ce^3+^-doped (Tb,Y)_3_Al_5_O_12_ single crystals grown by micropulling-down. Crystals were grown from melts with 0, 3, 6, 9, and 11 mol % Y_2_O_3_ deficiency. X-ray diffraction, electron probe microanalysis, and Raman spectroscopy revealed phase-pure garnet up to 3 mol % deficiency, rim-localized α-Al_2_O_3_ inclusions at 6 and 9 mol %, and core-localized perovskite-type inclusions at 11 mol %. Secondary-phase formation modified Tb/Y partitioning in the garnet matrix, affecting Ce^3+^ emission kinetics, Tb^3+^↔Ce^3+^ energy transfer, trap depth, and scintillation properties. The 11 mol % deficient crystal showed the highest thermal stability, with the Ce^3+^ thermal-quenching onset shifting from 375 to 425 K. The 9 mol % deficient crystal reached a luminous efficacy of 158 lm/W and a scintillation light yield of 33,600 photons/MeV. X-ray radiography using the 9 mol % Y_2_O_3_-deficient crystal showed clear images of an SD card. These results indicate that melt nonstoichiometry can tune the balance between photoconversion and scintillation performance in rare-earth garnets.

## Graphical abstract

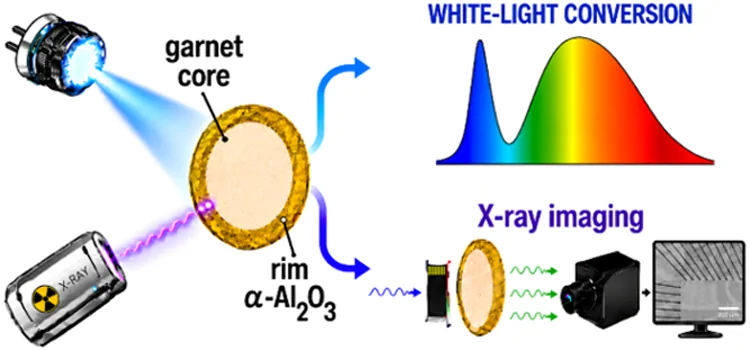

## 1 Introduction

The structural and chemical characteristics of insulating materials are impacted by crystal lattice distortions, point defects comprising atomic vacancies and interstitial constituents, as well as incorporations of secondary phase inclusions.^1–4^ Such defects modify the local structure and electronic structure of the insulating material.^5^ Understanding defects at the atomic scale is essential for tuning material properties.^6^ Accordingly, substantial effort has been devoted to developing computationally efficient methods to model defects, phase stability, and the simultaneous crystallization of immiscible phases in solids.^7,8^ In rare-earth aluminum garnets, antisite defects constitute an important class of intrinsic cation point defects, typically involving rare-earth cations on Al sites and, in some cases, Al cations on rare-earth sites. Specifically, yttrium aluminum antisite defects (*Y*_Al_^*x*^ ADs) arise when Y^3+^ ions incorrectly substitute for Al^3+^ ions within the crystal lattice.^9^ The origin of these defects is driven by thermodynamic conditions and stoichiometric imbalances during the crystal growth process.^7,10,11^ The *Y*_Al_^*x*^ antisite defects create shallow electron trapping centers, with trap depths varying between 0.0311 and 0.163 eV at temperatures ranging from 75 to 192 K.^12^ These shallow traps are of fundamental importance in melt-grown YAG and other garnet crystals, as they can trap excitons and thereby contribute to intrinsic ultraviolet emission.^13^ These shallow electron traps contribute to the substantial intensity of slow decay components in the scintillation response of the material, which is important for its performance in scintillating applications.^13,14^ Furthermore, Y_Al_^x^ antisite defects induce only localized perturbations of the crystal lattice. The associated strain fields modify the crystal-field environment of nearby luminescent ions, giving rise to inhomogeneous broadening and satellite structure of rare-earth spectral lines, while the defects themselves act as shallow electron traps.^10^ Although isolated antisite defects are too small relative to the wavelength of visible light to act as efficient scattering centers, light scattering in garnet-based photoconverters arises from extended structural inhomogeneities such as pores, refractive-index fluctuations, and secondary-phase inclusions and their interfaces. Such scattering redirects photons out of totally internally reflected trajectories, providing an escape pathway and thereby enhancing light extraction.^15,16^

Increasing the density of defects like oxygen vacancies, cation vacancies, or cation interstitials disrupts the ideal stoichiometric balance in a solid, leading to deviations from the ideal chemical formula and the formation of nonstoichiometric compounds. These compounds, known as Berthollides, have nonintegral atomic ratios unlike Daltonides.^17,18^ Thermodynamic considerations often impact the formation of nonstoichiometric compounds. The free energy may be diminished relative to the stoichiometric analog due to point defects that mitigate strain energy arising from ionic size mismatch and accommodate excess/deficient constituents. Reaction kinetics further influence stability, as the generation and migration kinetics of defects impact the equilibrium concentration and elemental homogeneity of the compound.^18,19^

Recent research has revealed that stoichiometric and nonstoichiometric materials exhibit markedly different luminescence characteristics. For instance, nonstoichiometric Yb^3+^,Er^3+^-codoped Y_3.2_Al_4.8_O_12_ transparent ceramics display fluorescence in the green spectral region, whereas stoichiometric Yb^3+^,Er^3+^-codoped Y_3_Al_5_O_12_ emits red light.^20^ A similar correlation was also observed for nonstoichiometric Ce^3+^,Nd^3+^-codoped Y_2.95_Al_5_O_12_ (Y-deficient) transparent ceramics, which displayed a high luminescence efficiency of ∼200 lm/W and thermal stability up to 680 K, compared to ∼130 lm/W efficiency and thermal stability to 380 K for stoichiometric Ce^3+^-doped Y_3_Al_5_O_12_ ceramics.^15^ Recently, moving away from stoichiometry was exploited to improve persistent luminescence in Ce^3+^, La^3+^-codoped Lu_3_Al_2_Ga_3_O_12_ single crystal giving over 10 h of afterglow.^21^ This enhanced durability of light emission after irradiation shows the potential of engineering nonstoichiometry to optimize luminescent performance. Investigations of nonstoichiometric Y_1 ± *x*_AlO_3_:Ce (*x* ≠ 0) perovskite single crystals revealed a 22% increase in light yield compared to stoichiometric counterparts.^22^ Furthermore, analysis of scintillation decay kinetics indicated a substantial reduction in the undesirable slow decay component.^22^

In the Tb_2_O_3_–Al_2_O_3_ oxide system, the perovskite phase TbAlO_3_ (TbAP) is thermodynamically the most stable and exhibits congruent melting behavior, meaning that it melts uniformly at its melting point without altering its composition.^23^ In contrast, the garnet phase Tb_3_Al_5_O_12_ (TbAG) undergoes incongruent melting. Upon melting, TbAG decomposes into a liquid phase with a composition different from the original garnet and a solid phase of TbAP, which remains the most stable phase in this system.^23,24^ The thermodynamic instability of the garnet phase, when coupled with deliberate deviations from stoichiometric Tb_2_O_3_: Y_2_O_3_: Al_2_O_3_ ratios, represents a novel methodological approach for crystallizing nonstoichiometric (Y-deficient) composite crystals. One previously unexplored class of nonequilibrium crystals exhibiting properties associated with composite structures beyond conventional phase diagram limitations. This integration enables multiphase systems to exceed the performance limitations of their parent structures while offering opportunities for property optimization. Single crystals are distinguished by their long-range atomic periodicity and defect-minimized lattice structures, which confer exceptional mechanical strength and optical transparency. Conversely, multiphase systems derive their functional advantages, including enhanced fracture toughness, thermal stability, and microstructural complexity, from the synergistic cosolidification of multiple phases. Therefore, multiphase crystals can be valuable for applications requiring both high performance and structural integrity, such as in advanced photonics, energy storage systems, and high-temperature structural materials.^25,26^ Furthermore, their composite-like nature allows for the tuning of properties through processing conditions, providing opportunities for innovation in material design.

## 2 Experimental Section

### 2.1 Crystal Growth Using the Micropulling-Down Technique

The Tb_2_O_3_, Y_2_O_3_, Ce_2_O_3_ and Al_2_O_3_ powders (99.99%, Iwatami Corp. Japan) were mixed in mole % presented in Table 1. The crystals were grown from the melt using the micropulling-down technique (μ-PD).^3,27^ Growth was carried out in an iridium crucible with a 3 mm inner diameter die, under an inert atmosphere of argon with purity 99.99%. A ⟨111⟩ oriented Y_3_Al_5_O_12_ seed crystal was used. The growth rate was maintained at 0.05 mm/min. For all characterization measurements, radial slices were extracted from the same axial position of each rod, located 15 mm from the seed end. This fixed extraction position was chosen because melt-grown multicomponent garnets, including crystals grown by the Czochralski and micropulling-down methods, exhibit progressive axial segregation along the growth direction. Consequently, the composition and properties of samples taken from the seed, middle, and tail regions differ.^28,29^ Sampling all crystals at the same axial position therefore ensures that the compositional and functional properties are compared under equivalent solidification conditions.

**Table 1:** Molar Compositions of Tb_2_O_3_, Y_2_O_3_, Ce_2_O_3_ and Al_2_O_3_ Used for the Crystallization of Multi-Phase Samples with Varying Degrees of Y_2_O_3_ Deficiency^a^ ^,^ ^b^

**sample ID**	**Y**_**2**_ **O**_**3**_ **deficiency** (mol %)	**Y**_**2**_ **O**_**3**_ (mol %)	^c^ **Tb**_**2**_ **O**_**3**_ (mol %)	**Al**_**2**_ **O**_**3**_ (mol %)	^d^ **Ce**_**2**_ **O**_**3**_ (mol %)
SC	0.0	11.5	25.0	62.5	1.0
NDY-3%	–3	8.5	25.0	62.5	1.0
NDY-6%	–6	5.5	25.0	62.5	1.0
NDY-9%	–9	2.5	25.0	62.5	1.0
NDY-11%	–11	0.5	25.0	62.5	1.0

a A stoichiometric reference sample (SC) is included for comparison. The abbreviation NDY denotes the degree of Y_2_O_3_ deficiency in the melt.

b For crystal growth.

c Tb_4_O_7_ and

d CeO_2_ powders were used

### 2.2 Crystal Phase Characterization

The crystalline phase purity of the grown crystals was examined by powder X-ray diffraction (PXRD) analysis. Measurements were performed using a Bruker D8 DISCOVER diffractometer equipped with a Cu Kα X-ray source (λ = 1.54 Å, *E* = 8.04 keV). Crystals were pulverized into fine powders utilizing an agate mortar. Continuous powder diffraction patterns were acquired between 20 and 65° (2θ) with a step size of 0.02°. Morphological and compositional characterization of crystals were carried out by Field Emission Scanning Electron Microscope (FE-SEM) (FEI Nova NanoSEM 230) coupled with an energy-dispersive X-ray spectrometer (EDS Apollo X silicon drift detector) with an energy resolution better than 135 eV, operated with Genesis EDAX microanalysis software (version 6.0). Prior to SEM-EDS measurements, samples were embedded in carbon resin (PolyFast, Struers) and pressed to obtain a large and flat surface. The samples were subsequently polished and coated with a thin gold layer using an Edwards Vacuum Auto 306 sputter coater. SEM imaging and EDS elemental mapping were performed by collecting secondary electron (SE) and characteristic X-ray signals, respectively.

The Electron Probe Microanalysis (EPMA) measurements were conducted to determine compositional properties using a Hitachi S-3400N SEM equipped with an Oxford Instruments INCA WAVE500 wavelength-dispersive X-ray spectroscopy (WDS) detector. A finely focused electron beam was directed onto the sample surface, exciting characteristic X-rays from the constituent elements. These X-rays were analyzed by wavelength-dispersive spectroscopy (WDS) to obtain high-precision quantitative data on elemental concentrations. The relative uncertainty of the analysis is approximately ±2% for Tb, Y, Al, and O and ± 10% for Ce, for which the concentration approaches the detection limit imposed by WDS counting statistics. These uncertainties were propagated to the stoichiometric coefficients reported in Table 2.

**Table 2:** EPMA Analysis of Radial Variations in the Elemental Composition of SC, NDY-9% and NDY-11% Crystals^a^

	**SC** single-phase	**NDY-9% nonstoichiometric**	**NDY-11% nonstoichiometric**
**distance (mm)**	actual composition	actual composition	actual composition
–1.2	Tb_2.21_Y_0.89_Ce_0.029_Al_4.87_O_11.87_	Tb_2.74_Y_0.28_Ce_0.023_Al_5.09_O_12.14_	Tb_2.05_Y_0.87_Ce_0.026_Al_4.75_O_11.40_
–0.6	Tb_1.93_Y_1.15_Ce_0.004_Al_4.90_O_11.88_	Tb_2.68_Y_0.36_Ce_0.009_Al_4.99_O_12.08_	Tb_1.84_Y_1.06_Ce_0.008_Al_4.74_O_11.45_
0	Tb_1.95_Y_1.18_Ce_0.003_Al_4.92_O_11.95_	Tb_2.62_Y_0.40_Ce_0.06_Al_4.87_O_11.96_	Tb_0.75_Y_0.29_Ce_0.01_Al_1.00_O_2.96_
0.6	Tb_1.98_Y_1.11_Ce_0.005_Al_4.15_O_11.92_	Tb_2.72_Y_0.36_Ce_0.08_Al_4.97_O_12.09_	Tb_1.94_Y_0.97_Ce_0.016_Al_4.72_O_11.46_
1.2	Tb_2.26_Y_0.80_Ce_0.034_Al_4.89_O_11.91_	Tb_2.79_Y_0.29_Ce_0.024_Al_5.07_O_12.12_	Tb_2.02_Y_0.86_Ce_0.030_Al_4.67_O_11.52_

a The relative uncertainty of the coefficients is ±2% for Tb, Y, Al, and O and ±10% for Ce, for example Al = 4.87 ± 0.10 and Ce = 0.029 ± 0.003

To further investigate the crystal purity and composition, micro-Raman spectroscopy (μ-RS) measurements were performed using an XploRA Plus HORIBA Scientific spectrometer equipped with an Olympus BX43 microscope. The spectral resolution was approximately 1 cm^–1^. An Open Electrode CCD camera with a multistage Peltier air-cooling system was used as the detector. The samples were positioned on a motorized XY stage. Measurements were performed at 300 K using a 785 nm (100 mW) laser light source. The laser beam intensity was precisely controlled at 10 mW through the application of neutral density filters of varying optical densities. The 50x long working distance objective lens was used. The spot size was approximately 10 μm^2^ for the 50× magnification. The integration time was set to 5 s with 10 accumulations in both cases.

### 2.3 Optical, Luminescence, Scintillation, and Thermoluminescence Properties

Optical absorption and photoluminescence excitation as well as emission spectra were acquired at 300 K using a Shimadzu 3101PC spectrophotometer and Edinburgh Instruments FLS1000 spectrofluorometer equipped with a xenon lamp as an excitation source. All recorded spectra were corrected for instrumentation artifacts. A pulsed nanoLED with 0.2 ns pulse duration and 40 MHz repetition rate facilitated time-resolved photoluminescence decay kinetics measurements. Temperature-dependent analyses between 12 and 650 K were performed using a closed-cycle cryostat coupled to the FLS1000 spectrofluorometer. Prompt luminescence decay curves were recorded by time-correlated single photon counting. The decay curves exhibited nonexponential behaviors and were fitted using the following eq 1:

1 $$I\left(t\right)=\sum_{i}I_{i}\exp\left(\frac{-t}{\tau_{i}}\right)+B$$

here, *I* represents luminescence intensity, *I*_*i*_ denotes the intensity at 0 ns, *t* signifies the time, τ_*i*_ is the decay time, and B stands for the time-independent background intensity. To quantify this, the effective decay time (τ_eff_) was calculated using eq 2:^2^

2 $$\tau_{\mathrm{eff}}=\frac{\sum_{i}I_{i}\tau_{i}^{2}}{\sum_{i}I_{i}\tau_{i}}$$

here, *I*_*i*_ represents the intensity, and τ_*i*_ stands for the decay time value of the *i*th component within the fit.

Photoconversion characterization was measured by positioning the crystalline specimen upon an illumination source equipped with a collimation lens to adapt the beam dimensions to the sample size. Excitation was provided by 450 nm radiation from a CNI laser diode and a 455 nm LED source. The optical power of the blue excitation was fixed at 1.0 W. Spectral acquisition was conducted utilizing a Gigahertz BTS-256LED spectrometer outfitted with an integrating sphere accessory.

Low-temperature (10–300 K) radioluminescence spectra (RL) and thermoluminescence (TL) glow curves were obtained with a custom setup built with an Inel Cu-anode X-ray generator, an ARC SP-500i monochromator, a Hamamatsu R928 photomultiplier and an APD Cryogenics helium cooler controlled by a Lake Shore 330 unit. TL measurements were performed between 10 and 300 K at a heating rate of 0.14 K/s, following a 10 min X-ray excitation. RL spectra were recorded starting at 300 K and terminating at 10 K, which was aimed at avoiding a possible contribution from the potential thermal release of charge carriers to the RL yield. The low-temperature glow curves obtained from thermoluminescence measurements were analyzed using quasi-continuous distributions of trap levels. In this approach, a single trap does not correspond to a discrete energy value but rather spans a range of energy values, as determined by the number of Gaussian functions used in the fitting process.^30,31^ High-temperature (300–720 K) thermoluminescence experiments were carried out using a Lexsyg Research fully automated TL/OSL reader (Freiberg Instruments GmbH). A VF-50J X-ray lamp with a W anode operated at 12 kV and 0.1 mA was used as the excitation source. Thermoluminescence glow curves were recorded using a 9235QB-type photomultiplier from ET Enterprises in the range of 300–723 K with a heating rate of 5 °C/s. All experiments were controlled through LexStudio 2 software. The glow curves were deconvoluted into TL components, using GlowFit software.^32^

To measure the scintillation light yield and decay time, the crystals were enclosed in several layers of Teflon tape and optically coupled to the light entrance window of the R7600 photomultiplier tube (PMT; Hamamatsu) using an optical grease. The high voltage was provided by the ORTEC 556 power supply, and signals were extracted from the PMT anode. These signals then passed through 10 μs shaping amplifier, the ORTEC 570, before being digitized by the Pocket MCA 8000A multichannel analyzer (MCA; Amptek Co.). Scintillation decay curves were obtained using a digital oscilloscope (Tektronix TDS3034B) under excitation by 662 keV photons from the ^137^Cs radioisotope.

The uncertainties of the photoluminescence and scintillation decay components correspond to the standard errors of the multiexponential fits (eq 1), and the uncertainty of the effective decay time was obtained by error propagation through eq 2. The light yield and energy resolution uncertainties were derived from the standard errors of the Gaussian fits of the full absorption peaks in the pulse height spectra. The parameters of the shallow trap distributions (Table S1) carry a relative uncertainty of ± 5%. The trap activation energies obtained from deconvolution of the high temperature glow curves (Table S2) carry an uncertainty of ± 0.05 eV and the frequency factors are reliable within a factor of 3, reflecting the figure of merit of 5% and the correlation between both parameters in the fit. The uncertainties of the colorimetric parameters are ± 50 K for CCT, ± 2 for CRI, and ± 5% relative for LE, based on the calibration of the spectroradiometer.

### 2.4 Electron Paramagnetic Resonance

Electron paramagnetic resonance measurements were conducted on a commercial Bruker EMXplus spectrometer operating at the X band (9.4 GHz) and TE011 mode. The measurements were performed at 20 K using an Oxford Instruments ESR900 cryostat. The sample was placed in a quartz tube for the measurements. X-ray irradiation of samples in the quartz tube was conducted at 20 K using ISO–DEBYEFLEX 3003 highly stabilized X-ray equipment for structural analysis (tungsten X-ray tube, 50 kV, 30 mA).

## 3 Results and Discussion

### 3.1 PXRD, SEM-EDS, EPMA and Micro-Raman Analysis

Figure 1A presents the as-grown crystal rods and polished cross sections of SC, NDY-3%, NDY-6%, NDY-9%, NDY-11% crystals. For clarity and conciseness, the crystals are referred to by their abbreviations as listed in Table 1. The surfaces exhibit opaque, rough morphologies attributable to thermal etching and the formation of surface defects during growth.^33^ The SC exhibits a uniform, rod-like morphology, which is indicative of congruent melting and crystallization under stoichiometric conditions. In contrast, crystals grown from Y_2_O_3_-deficient melts exhibit a tapered, conical termination at the tail region. This distinct morphological feature arises from the progressive depletion of constituent components during solidification, reflecting the change in the dynamics of phase separation. Such depletion mechanisms significantly influence the nucleation rate under conditions of constant pulling speed. Figure 1B presents the powder X-ray diffraction patterns acquired from the SC, NDY-3%, NDY-6%, NDY-9%, and NDY-11% crystals. The SC crystal retains a garnet structure, as verified by comparison to reference data (#PDF 76-0111). This SC crystal is indexed to the cubic *Ia*3̅*d* space group (No. 230) without detectable secondary phases. The PXRD patterns of NDY-3% and NDY-6% samples reveal no additional phases apart from the garnet phase. However, the sensitivity of the XRD technique for detecting secondary phases in garnet-type structures is limited to approximately 2%.^4,34^ As a result, the presence of secondary phases with concentrations below this threshold may remain undetected.

**Figure 1:**
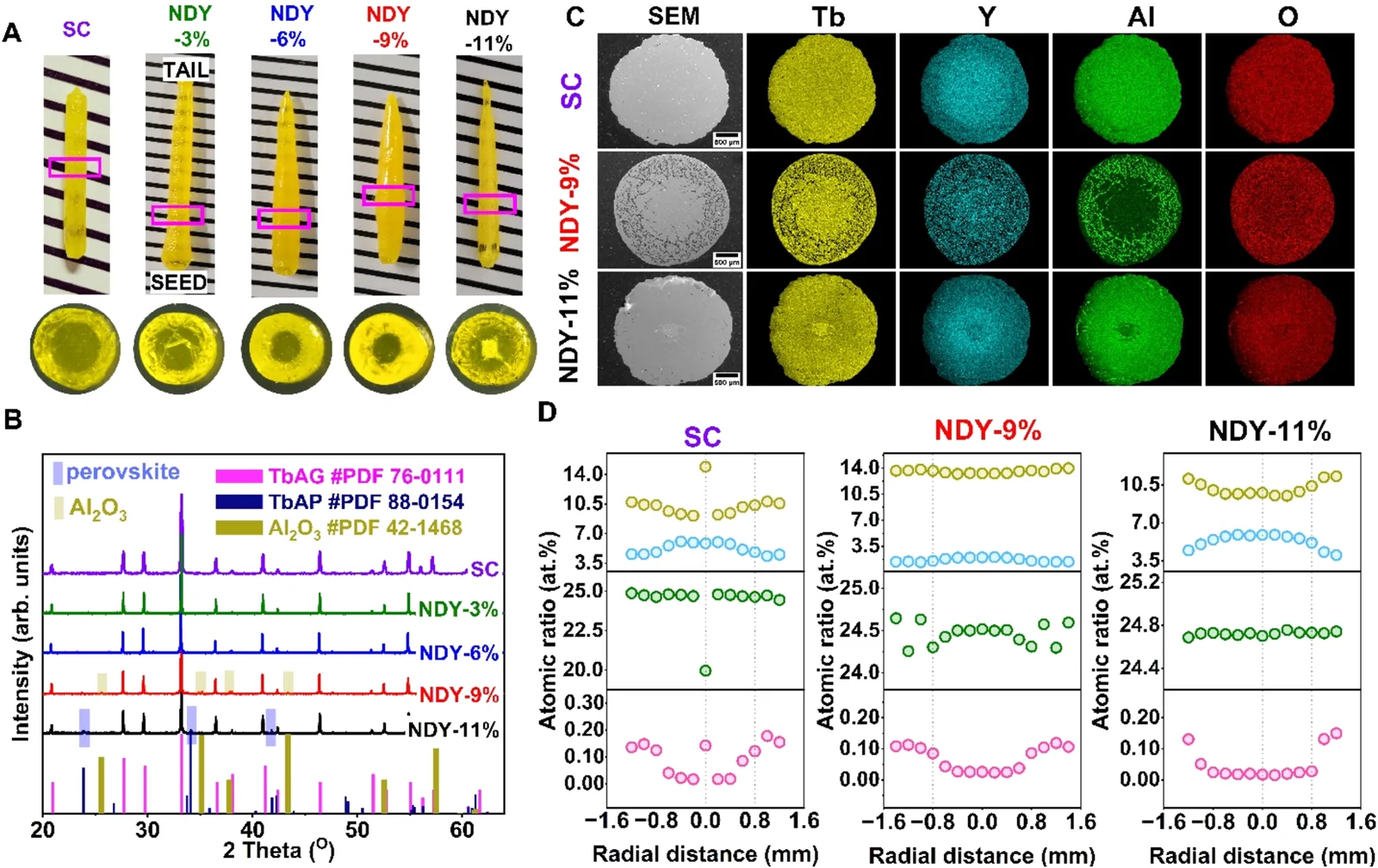
(A) As-grown rods of the single-phase garnet (SC) and mixed-phase (NDY-9% and NDY-11%) crystals with polished radial cross sections. (B) PXRD patterns of the SC, NDY-9% and NDY-11% crystals, compared with reference patterns for Tb_3_Al_5_O_12_ garnet (TbAG, #PDF 76-0111), TbAlO_3_ perovskite (TbAP, #PDF 88-0154), and α-Al_2_O_3_ (#PDF 42-1468). (C) EDS elemental maps of terbium (yellow), yttrium (cyan), aluminum (green), and oxygen (red). (D) EPMA radial atomic ratio profiles of Tb, Y, Al and Ce for SC, NDY-9% and NDY-11% crystals. The EPMA uncertainty of ± 2% relative is smaller than the symbol size.

These results suggest that low degrees of Y_2_O_3_ deficiency may lead to the formation of secondary phases at concentrations below the detection limit. This observation is consistent with previous studies that have reported the successful incorporation of stoichiometric variations into the garnet structure without the formation of significant secondary phases.^35^ In contrast, the PXRD analysis of NDY-9% revealed the presence of a secondary α-Al_2_O_3_ phase, in addition to the garnet phase, with a phase fraction of 4.78 ± 0.5% as determined by Rietveld refinement (see Figure S1 in the Supporting Information (SI)). Similarly, in the NDY-11% sample, a perovskite phase is identified with a phase fraction of 3.91 ± 0.5% (Figure S1). The diffraction patterns from the beginning and middle sections show minor differences, whereas the end section exhibits more pronounced deviations, consistent with segregation accumulating toward the end of solidification (Figure S1A).^28^ The phase constitution reported in Figure 1 is therefore representative of the near-seed region from which all characterized slices were extracted.

Scanning electron microscopy coupled with energy dispersive X-ray spectroscopy was used to investigate the radial phase distribution of garnet, perovskite, and α-Al_2_O_3_ phases in SC, NDY-3%, NDY-6%, NDY-9%, and NDY-11% crystals (see Figures 1C and S2). The SC revealed a heterogeneous distribution of Y^3+^ and Tb^3+^ cations, an effect characteristic of garnets containing cations with differing ionic radii.^27,36^ Microstructural analysis of NDY-9% and NDY-11% revealed distinct spatial segregation of the secondary phases, governed by divergent solidification pathways. In NDY-9%, a well-defined lamellar eutectic-type microstructure, comprising alternating garnet and garnet-Al_2_O_3_ phases, is observed to preferentially nucleate at the crystal rim. This rim-localized eutectic growth suggests rapid interfacial undercooling at the crystallization front, favoring coupled garnet-Al_2_O_3_ phase formation under high thermal gradient conditions. This observation is consistent with the phase diagram, which indicates that Y_3_Al_5_O_12_–Al_2_O_3_ eutectic structures crystallize at lower temperatures than single-phase regions. As the rim of the crystal experiences lower temperatures compared to the core during growth, the formation of secondary phases such as α-Al_2_O_3_ at the outer edges is thermodynamically favored.^37^ This observation is further supported by previous studies that revealed the phases distribution dependence on the solidification rate.^26^ The pronounced Tb enrichment observed at the center of the NDY-11% cross-section in the EDS map (Figure 1C) corresponds to the Tb-rich perovskite phase.^37^ The ABO_3_ perovskite stoichiometry contains a higher rare-earth cation fraction (50%) than the A_3_B_5_O_12_ garnet (37.5%), causing the strong Tb contrast in the crystal core. The formation of a rare-earth-rich phase from a Y_2_O_3_-deficient melt may appear counterintuitive. It should be noted, however, that the Tb_2_O_3_ concentration is held constant at 25.0 mol % throughout the series (Table 1). For NDY-11% the total rare-earth oxide content is 29.8 mol % against 70.2 mol % α-Al_2_O_3_, compared with 37.5:62.5 for garnet stoichiometry. The melt is therefore α-Al_2_O_3_-rich, rather than significantly depleted in rare-earth oxides, and a minor phase with a 3.91% phase fraction imposes no mass-balance constraint. The principal effect of the Y_2_O_3_ deficiency is a change in the composition of the rare-earth sublattice, with the Y/(Y+Tb) ratio decreasing from 31.5% for SC to approximately 2.0% for NDY-11%. The confinement of this phase to the core arises from two coupled factors. First, the system thereby approaches the binary Tb_2_O_3_–Al_2_O_3_ system, in which the garnet phase melts incongruently and the perovskite TbAlO_3_ is the thermodynamically most stable, congruently melting phase.^23,24^ Under these conditions, perovskite becomes the primary crystallizing phase, while the garnet-stabilizing effect of Y is largely suppressed. Second, the radial thermal gradient inherent to the μ-PD method makes the central region of the solid–liquid interface the hottest part of the growing crystal, hence the high-temperature primary perovskite phase nucleates preferentially in the core. Its crystallization locally consumes rare-earth cations and leaves the surrounding liquid further enriched in α-Al_2_O_3_, from which the garnet matrix subsequently solidifies in the cooler peripheral region.^37^ This finding is independently corroborated by the EPMA composition of the crystal center, Tb_0.75_Y_0.29_Ce_0.01_Al_1.00_O_2.96_ (Table 2), which matches an ABO_3_ cation ratio, and by the disappearance of Raman activity above 600 cm^–1^ in the core region (section 3.2). It should be noted that the EDS maps in Figure 1C display qualitative X-ray intensity distributions, whereas quantitative compositions were determined by EPMA (Figure 1D, Table 2). The quantitative Y profile in Figure 1D increases from ∼4.6 atom % at the rim to a broad central maximum of ∼5.8–6.0 atom %, with only a marginal decrease at the core position identified as perovskite by the concurrent decrease in Al (∼24.5 → ∼20 atom %) and increase in Tb (∼10 → ∼15 atom %). This behavior reflects two opposing contributions: the higher rare-earth cation fraction of the perovskite phase raises the Y atomic fraction, while preferential incorporation of the larger Tb^3+^ ions into the perovskite lattice reduces Y relative to the adjacent garnet.

Radial compositional variation in the SC, NDY-9%, and NDY-11% crystals was evaluated by EPMA at 0.6 mm intervals across the crystal diameter (Table 2). Among the three samples, NDY-9% shows the smallest radial variation in Al, with an average EPMA-derived Al coefficient of 5.00 ± 0.09, consistent with a compositionally stable garnet matrix across the analyzed section. The SC crystal also remains close to the garnet composition at most measured positions, although one isolated low-Al value (Al = 4.15) broadens the apparent spread in composition. In contrast, NDY-11% exhibits a pronounced core-rim compositional discontinuity. Whereas the outer positions retain garnet-like compositions, the crystal center shifts abruptly to Tb_0.75_Y_0.29_Ce_0.01_Al_1.00_O_2.96_. This composition departs strongly from the A_3_B_5_O_12_ garnet stoichiometry and approaches an ABO_3_-type cation ratio, indicating that the crystal center corresponds to a chemically distinct phase and not to a compositionally perturbed garnet region. Together with the PXRD, EDS, and EPMA results discussed above, these data support assignment of a perovskite phase in the core of NDY-11%, surrounded by a garnet-rich outer region.

### 3.2 Raman Spectroscopy

Micro-Raman spectroscopy was used to probe local lattice distortions and strain associated with secondary-phase inclusions. Garnet crystals adopt the cubic structure with space group *Ia*3̅*d* and, in principle, exhibit 25 Raman-active modes.^38^ Figure 2 compares Raman spectra acquired between 100 and 1000 cm^–1^ along radial line scans from the rim to the crystal center for the SC, NDY-9%, and NDY-11% samples. Spectra for the remaining compositions are provided in Figure S3. Of the 25 symmetry-allowed modes, 14 were experimentally resolved. These modes are characteristic of a (Tb,Y)_3_Al_5_O_12_ garnet lattice and include the low-frequency vibrations associated with translational motion of the rare-earth sublattice. For all crystals, Raman spectra acquired from inclusion-free matrix regions are consistent with a mixed TbAG-YAG garnet solid solution.^39^ the Raman spectra from the core of the NDY-11% crystal (Figure 2C) provide further evidence of the formation of a perovskite secondary phase. While several bands at approximately 107, 257, 331, 390, 417, and 543 cm^–1^ overlap with those of the surrounding (Tb,Y)_3_Al_5_O_12_ garnet matrix, all Raman activity above 600 cm^–1^ disappears in the core region, in marked contrast to the garnet lattice response. This distinction is critical because orthorhombic XAlO_3_ perovskites (X = Y, Tb) are reported to display strong Raman modes only within the 100–580 cm^–1^ spectral range, with no significant higher-wavenumber features.^40^ The suppression of the high-frequency part of the spectrum therefore provides clear spectroscopic evidence that the central region of the NDY-11% crystal contains a perovskite phase. This conclusion agrees with the phase analysis derived independently from PXRD and EPMA.^40^ The NDY-9% crystal remains predominantly single-phase garnet on the submillimeter scale, displaying the expected lattice modes at ∼200–400 cm^–1^, AlO_6_ bending/stretching modes at ∼500–650 cm^–1^, and AlO_4_ stretching modes at ∼800–900 cm^–1^. Relative to typical YAG-like spectra, these bands are slightly blue-shifted, particularly in the 600–700 cm^–1^ region, indicating local compositional and strain-induced perturbations of the garnet lattice. At higher spatial resolution, however, linear Raman scans resolve an additional sharp band at ∼418 cm^–1^ near the rim and rim-center regions of NDY-9%. This feature is consistent with the presence of the α-Al_2_O_3_ phase, demonstrating that these inclusions are spatially localized in this composition.^41^ No corresponding α-Al_2_O_3_ phase was detected in NDY-3%, either near the rim or in the crystal interior. In contrast, NDY-6% shows a localized α-Al_2_O_3_ signature in the rim and rim-center regions (Figure S3).

**Figure 2:**
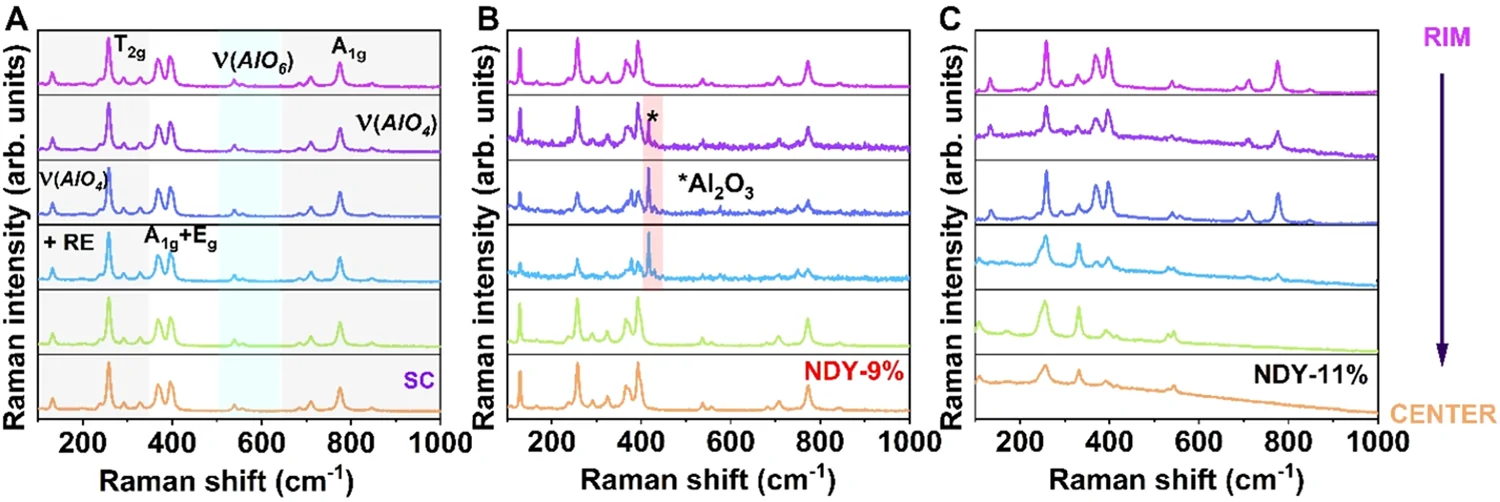
Raman spectra collected along a linear scan from the rim to the center of the (A) SC, (B) NDY-9% and (C) NDY-11% crystals.

### 3.3 Thermoluminescence Analysis

Thermoluminescence glow-curve measurements show that Y_2_O_3_-deficiency in the melt, together with secondary-phase formation, strongly influences charge-trap distribution, their depth, and thermal stability. Figure 3A.B present the low-temperature (10–300 K) and high-temperature (300–550 K) thermoluminescence glow curves of the SC, NDY-9%, and NDY-11% crystals, respectively, whereas the corresponding TL curves for NDY-3% and NDY-6% are shown in Figure S4. All TL measurements were performed under identical conditions, ensuring that the recorded intensities are directly comparable on an absolute scale.^42^ The low-temperature TL glow curves exhibit a multipeak structure with partially overlapping maxima over 40–250 K (Figures 3A and S4), indicating the presence of multiple shallow trapping states. TL maxima in this region are commonly associated with electron traps related to *Y*_Al_^*x*^ antisite defects.^43^ By contrast, the origin of the features above 200 K is less straightforward and may involve tunneling-mediated processes, a distribution of trapping states, and trace impurities such as Yb, Cr, Fe, or Mn.^44,45^

**Figure 3:**
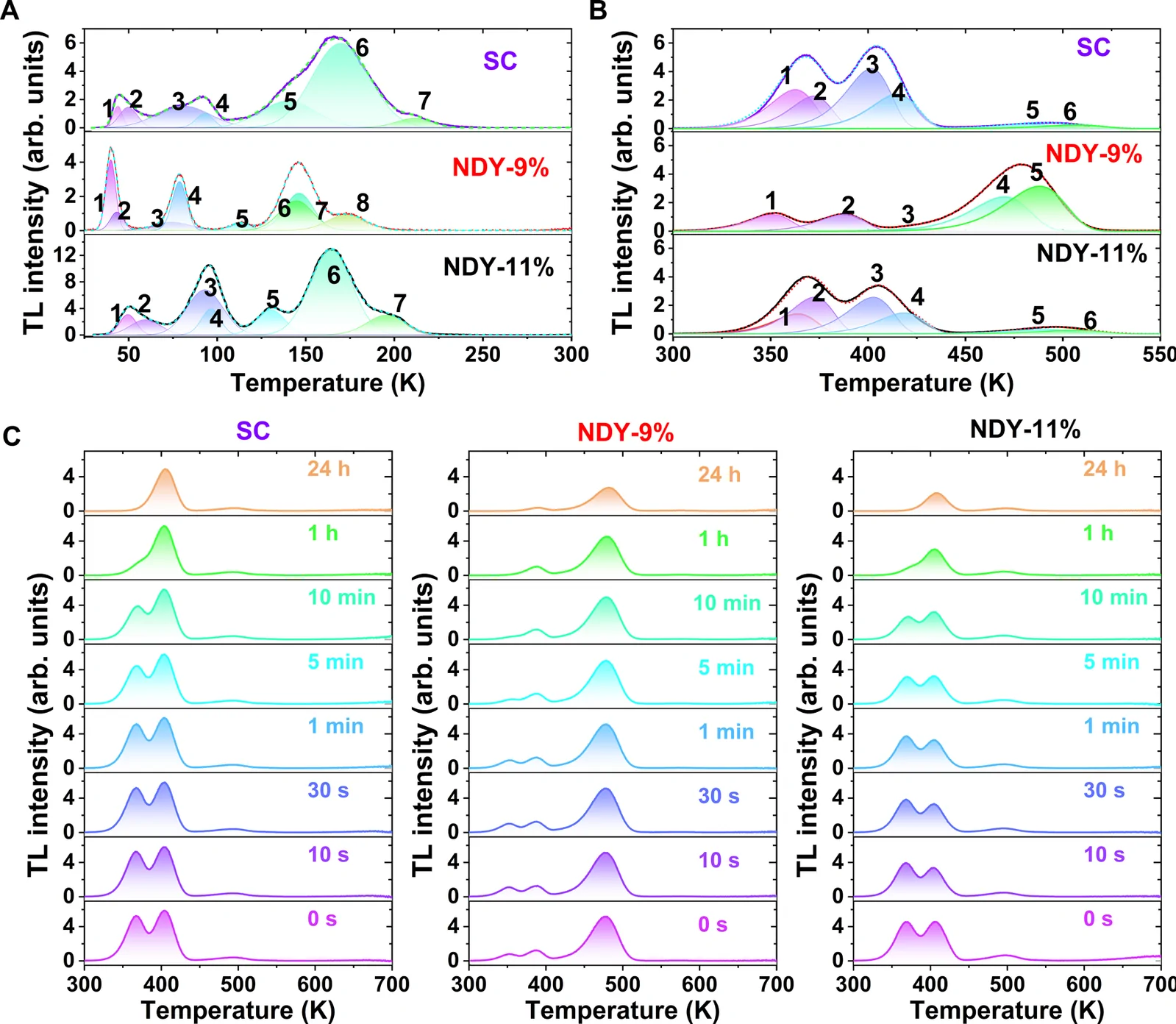
Thermoluminescence glow curves of SC, NDY-9% and NDY-11% crystals recorded after X-ray irradiation over (A) 10–300 K, (B) 300–550 K and (C) time-dependent thermoluminescence curves between 0 s and 24 h after irradiation. Each glow curve was deconvoluted into individual peaks to resolve individual trap contributions.

The high-temperature TL glow curves similarly exhibit overlapping bands over 300–550 K (Figures 3B and S4), consistent with multiple deeper traps. The bands in the 320–530 K range are commonly related to oxygen vacancies *(V*_*O*_^*••*^ *)* and may also be affected by trace impurities such as Yb, Cr, or Mn.^27,45–47^ The TL glow curves are broadly conserved across the series, while the relative intensities and shifts in peak positions vary with the degree of Y_2_O_3_-deficiency. These systematic variations correlate with the Tb/Y ratio on the rare-earth sublattice of the garnet phase (Figures 1, S2 and Table 2) and, accordingly, with the secondary-phase formation that influences rare-earth partitioning during crystal growth. SC and NDY-11% exhibit similar low- and high-temperature TL profiles, consistent with comparable Tb and Y concentrations in their garnet phase, whereas NDY-3%, NDY-6%, and NDY-9% form a second group with similar TL responses associated with pronounced Tb enrichment in the garnet phase. These observations indicate that rare-earth sublattice chemistry, particularly the Tb/Y ratio, strongly influences the population and energetic distribution of shallow and deep traps by modifying the local environment of intrinsic trapping centers in Ce-doped aluminum garnets.

To better resolve the trap states active between 10 and 300 K, the glow curves were analyzed using a quasi-continuous distribution of trap levels. In this approach, traps are not treated as discrete energy levels but as an energy distribution represented by a series of Gaussian functions in the fitting procedure. This formalism provides a more realistic description of the trap population and improves the precision and interpretability of the glow-curve analysis.^30,48^ Trap states active in the 300–550 K temperature range were analyzed by deconvolution of the TL glow curves into individual components using the GlowFit program.^32^ A satisfactory fit, with a figure of merit of 5%, required up to six components with distinct trap energy. The extracted trap parameters are summarized in Tables S1 and Table S2. Time-dependent TL glow curves recorded in the 300–550 K temperature range after X-ray irradiation (Figures 3C and S4C, S4D and S4E) provide further insight into trap stability at room temperature. In this analysis, the rate at which a given glow-curve component fades during storage is governed by the trap depth and frequency factor, whereas its intensity reflects the concentration of the corresponding traps. These two quantities are therefore considered separately in the following discussion. For all examined crystals, the TL intensity decreases with increasing delay time from 0 s to 24 h, with the strongest fading occurring in the lower-temperature region of the glow curve. This behavior is consistent with thermally activated release of carriers from moderately deep traps during storage at room temperature, whereas deeper traps retain charge for longer times and continue to contribute to TL after extended delays.

Clear composition-dependent differences are observed. In SC, the TL response is dominated by bands in the 360–420 K temperature range, which decay substantially within minutes to hours, while the contribution at 480–520 K remains weak at all delay times. In contrast, NDY-9% exhibits a pronounced broad band at 480–520 K that persists up to 24 h, whereas the lower-temperature features fade rapidly. The position of this band at 480–520 K identifies the underlying traps as deep and therefore thermally stable at room temperature, while its large integrated intensity indicates that such traps are present in comparatively high concentration in NDY-9%. Its detectability after 24 h reflects both factors: the trap depth determines the fading rate, whereas the initial concentration determines how long the residual signal remains above the detection limit. NDY-11% shows an intermediate response. The 360–420 K-band remains clearly visible at short delay times and decays during storage, whereas the 480–520 K contribution is markedly weaker than in NDY-9% and falls below the detection limit at shorter delay times, indicating a lower concentration of deep traps and not a lower thermal stability of the individual trapping centers. The corresponding time-dependent TL curves of NDY-3% and NDY-6% (Figure S4C) show behavior similar to that of NDY-9%, including a persistent high-temperature contribution. The contrasting fading behavior correlates with the secondary-phase constitution. NDY-6% and NDY-9% contain α-Al_2_O_3_ inclusions, which introduce garnet-corundum heterointerfaces likely associated with a high density of structural defects, such as misfit-related dislocations and related point-defect complexes. Such defect-rich interfacial regions may stabilize deep trapping centers.^49^ In NDY-3%, α-Al_2_O_3_ was not detected by either PXRD or Raman spectroscopy, yet its time-dependent TL response follows that of NDY-6% and NDY-9%. This similarity is consistent with the comparable Tb enrichment of the garnet phase in these three crystals (Table 2, Figure S2). In Ce-doped garnets, deep traps and nonradiative recombination pathways have also been linked to oxygen-vacancy-related defects and their complexes, which is consistent with the dominant long-lived band at 480–520 K observed for NDY-9% and, more generally, for the α-Al_2_O_3_-bearing crystals.^50^ By contrast, NDY-11% contains a perovskite secondary phase, implying a different partitioning of constituents and a different interfacial defect chemistry. The lower intensity of the 480–520 K-band in this sample is therefore consistent with a lower concentration of deep traps in the garnet phase. The trap parameters obtained from the glow-curve deconvolution (Table S2) confirm that this difference does not originate from a lower thermal stability of the individual trapping center. The activation energies of the two components comprising this band are 1.28 and 1.32 eV for NDY-9% and 1.32 and 1.38 eV for NDY-11%, identical within the fitting uncertainty of ± 0.05 eV, while the corresponding frequency factors decrease from 17.9 × 10^14^ and 13.8 × 10^14^ s^–1^ to 0.27 × 10^14^ and 0.37 × 10^14^ s^–1^, a reduction far exceeding the fitting uncertainty. The deep traps in NDY-11% are therefore at least as stable at room temperature as those in NDY-9%, and the weaker signal reflects a smaller population of these centers in the garnet matrix, together with a shift in the energetic position of vacancy-related traps upon perovskite formation.^51^ The time-dependent TL data indicate that NDY-3%, NDY-6%, and NDY-9% contain a larger fraction of deep, room-temperature-stable traps than the perovskite-bearing NDY-11% crystal, whereas SC is dominated by intermediate-temperature traps that decay more rapidly.

### 3.4 Temperature Dependence of PL Characteristics

Figure 4A compares the room-temperature excitation spectra monitored at 567 nm, corresponding to Ce^3+^ ions emission, for the single-phase and multiphase crystals. All samples show two main excitation bands centered at approximately 325 and 480 nm, assigned to the parity-allowed 4f → 5d_2_ and 4f → 5d_*1*_ transitions of Ce^3+^ ions.^52–55^ In the 300–350 nm spectral region, these Ce^3+^ excitation features overlap with spin-forbidden 4f^8^ → 4f^7^5d^1^ transitions of Tb^3+^ ions. Additional bands at approximately 220 and 275 nm are attributed to the spin-allowed low-spin 4f → 5d_2_ and 4f → 5d_*1*_ transitions of Tb^3+^ ions, respectively.^52–56^ The spectra also contain Tb^3+^-related lines at 340, 346, 354, 360, and 370–385 nm, assigned to the ^7^F_6_ → ^5^L_8_+^5^L_7_+^5^G_2_, ^7^F_6_ → ^5^G_3_, ^7^F_6_ → ^5^L_6_, ^7^F_6_ → ^5^G_5_, ^7^F_6_ → ^5^G_6_ transitions, respectively.^52,53,57^ The presence of Tb^3+^-related excitation features within the Ce^3+^ excitation spectra, together with their substantial spectral overlap, is consistent with Tb^3+^→Ce^3+^ energy transfer.^52,53,55^ Figure 4B compares the Ce^3+^ emission spectra recorded under 440 nm excitation for the single-phase and multiphase crystals. All spectra exhibit the characteristic Ce^3+^ 5d_1_ → 4f emission doublet (^2^F_5/2_, ^2^F_7/2_) evidencing the successful incorporation of Ce^3+^ ions into the cubic garnet lattice. Temperature-dependent excitation and emission spectra measured between 12 and 500 K are shown in Figure S5. Figure 4C presents the excitation–emission matrix (EEM) of the NDY-11% crystal and shows that Ce^3+^ emission can be excited efficiently over a broad wavelength range from 230 to 480 nm. This excitation range reflects the coexistence of direct Ce^3+^ 4f → 5d excitation and indirect Tb^3+^→Ce^3+^ energy transfer sensitization, in agreement with the Tb^3+^-related excitation features detected between 350 and 380 nm. The relatively uniform EEM intensity across this excitation window indicates that Ce^3+^ emission remains efficient over a wide range of excitation wavelengths, despite the presence of secondary phases. Figure 4D shows that the temperature at which the integrated Ce^3+^ emission remains at 90% of its room-temperature value (T_90_) varies nonmonotonically with nominal Y_2_O_3_-deficiency. T_90_ decreases slightly from SC (∼375 K) to NDY-3% (∼365 K) and NDY-6% (∼360 K), returns to the SC value for NDY-9% (∼375 K), and increases markedly for NDY-11% (∼425 K). This trend correlates, at least in part, with the Tb/Y ratio in the garnet phase. As shown in Table 2 and Figure S2, the garnet phase in NDY-3%, NDY-6%, and NDY-9% is Tb-enriched relative to SC, whereas NDY-11% has a Tb/Y ratio closer to that of SC. Such changes in rare-earth sublattice composition are expected to modify the local environment of Ce^3+^ ions and the density of Tb–Ce interaction pairs. Enrichment in the larger Tb^3+^ ions may perturb the crystal-field environment and the nephelauxetic effect acting on the Ce^3+^ 5d manifold, thereby shifting the lowest *5d*_*1*_ state and modifying the activation barrier for nonradiative 5d_1_ → 4f relaxation in terms of the configuration-coordinate framework.^2,34^ At the same time, the difference in T_90_ between SC and NDY-11%, despite their similar Tb/Y ratios, indicates that rare-earth sublattice composition alone cannot explain the observed thermal stability. An additional microstructure-dependent contribution is therefore likely. This explanation is consistent with the established role of traps and defect-mediated recombination pathways in Ce^3+^-doped garnets, where antisite disorder, oxygen-vacancy-related defects, and vacancy-associated complexes can compete with radiative emission once carriers become thermally activated.^58^ In the present system, increasing Y_2_O_3_-deficiency drives the garnet lattice away from ideal stoichiometry and is therefore expected to modify the defect chemistry of the matrix, including the populations of cation vacancies, antisite defects, and vacancy-related complexes, as discussed in Section 3.3. Secondary-phase formation may further influence thermal stability through two coupled effects: nonradiative recombination at inclusion-matrix interfaces and redistribution of defects in the garnet matrix as part of the off-stoichiometry is accommodated by precipitation of a competing phase. For the α-Al_2_O_3_-containing samples (NDY-6% and NDY-9%), the corundum inclusions are structurally dissimilar to the garnet host and are therefore likely to form incoherent, strain-inducing interfaces with defect-rich interfacial regions that can act as nonradiative sinks.^4,59^ By contrast, the NDY-11% sample contains a perovskite secondary phase, which is chemically and structurally closer to the garnet matrix than corundum implying a different interfacial defect chemistry that may reduce extrinsic nonradiative losses and contribute to the higher T_90_ observed for this sample.^26,34^ This points to a different phase-selection regime from that operating in the α-Al_2_O_3_-bearing samples. Perovskite formation may also accommodate part of the off-stoichiometric composition outside the garnet lattice, thereby reducing the extent to which the compositional imbalance is retained as point defects in the garnet phase. This observation is consistent with a lower concentration of antisite- and vacancy-related defects in the garnet matrix and, consequently, with weaker defect-assisted nonradiative recombination. Figure 4E,F show that Ce^3+^ emission kinetics change systematically with increasing Y_2_O_3_-deficiency, consistent with changes in Tb↔Ce energy exchange within the garnet phase.^52,54,60,61^ The decay curves in Figure 4E are nonexponential and were therefore analyzed using a multiexponential function, with the results summarized by an effective decay time (τ_eff_). As Y_2_O_3_-deficiency increases, τ_eff_ rises from ∼170 ns (±5 ns) for SC to 198 ns (±7 ns) for NDY-3%, 300 ns (±12 ns) for NDY-6%, and 423 ns (±15 ns) for NDY-9%, and then decreases to 262 ns (±10 ns) for NDY-11%. All these values are substantially longer than the prompt Ce^3+^ decay typically reported for Ce-doped garnets (50–60 ns), indicating a pronounced delayed contribution to the Ce^3+^ emission.^15,61^ EPMA data (Table 2) show that these kinetic changes correlate with the composition of the garnet phase. SC remains close to nominal rare-earth occupancy, with Tb = 1.93–2.26 and Y = 0.80–1.18, whereas NDY-9% is strongly Tb-enriched, with Tb = 2.62–2.79 and Y = 0.28–0.40. By contrast, NDY-11% shows intermediate values (Tb = 1.84–2.05, Y = 0.86–1.06) that are much closer to SC. This compositional trend is consistent with a change in the rate of bidirectional Tb↔Ce energy transfer.^62^ In particular, the dense Tb sublattice in NDY-9% is expected to strengthen Tb-mediated forward and back energy-transfer cycles, thereby increasing the delayed component and prolonging τ_eff_. The shorter τ_eff_ in NDY-11% is consistent with both the lower Tb content in the garnet phase and the microstructural transition to perovskite-containing crystals, which together reduce the effective density of Tb–Ce interaction pairs. The temperature dependence shown in Figure 4F further supports this interpretation. For all compositions, τ_eff_ increases with temperature, reaches a broad maximum in the intermediate-temperature range (∼350–450 K), and then decreases at higher temperature. The peak magnitude follows the order NDY-9% (∼400 ns) > NDY-11% (∼250–280 ns) ≳ NDY-6% (∼220–240 ns) > NDY-3% ∼ SC (∼180–200 ns). This nonmonotonic behavior is consistent with thermally assisted Tb–Ce resonance exchange, which becomes more efficient on heating and increases the delayed contribution to Ce^3+^ emission. Above ∼400–450 K, the subsequent decrease in τ_eff_ indicates the onset of thermally activated nonradiative relaxation, which progressively competes with radiative recombination. The role of phonon-assisted Tb→Ce transfer is further supported by the temperature dependence of the photoluminescence intensity under excitation into the 5d_1_ state of Tb^3+^ ions at 270 nm (Figure S6). Although trap-related processes may also affect the Ce^3+^ decay, as indicated by the thermoluminescence data, the strong correlation between τ_eff_ and the Tb concentration of the garnet phase indicates that Tb↔Ce energy transfer is the dominant factor shaping the kinetic trends in Figure 4E,F. In this context, secondary-phase formation acts mainly indirectly by modifying rare-earth partitioning and, consequently, the composition of the garnet phase during crystal growth. Figure S7 shows the Ce^3+^ decay curves measured at selected temperatures between 12 and 500 K.

**Figure 4:**
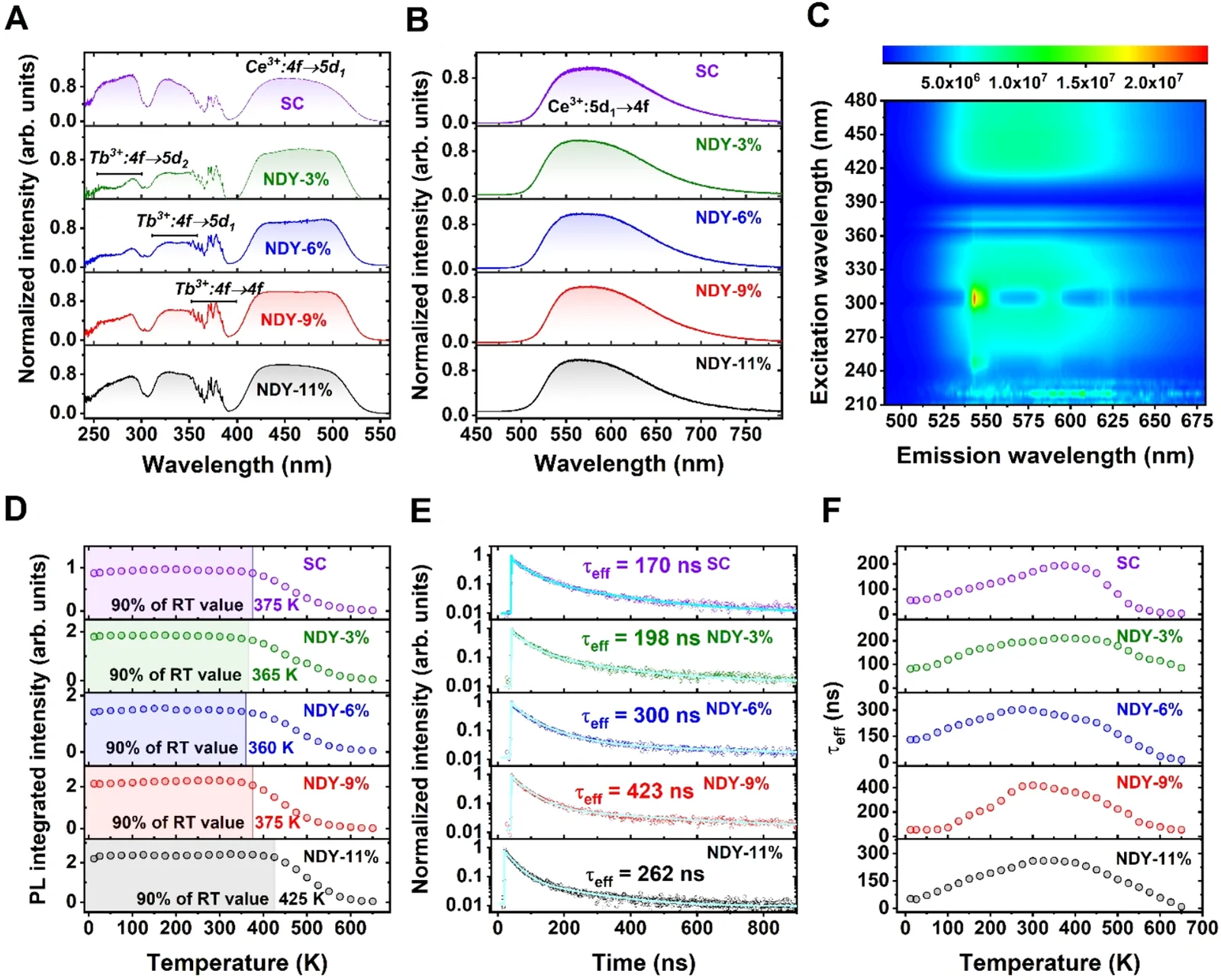
(A) RT excitation spectra of Ce^3+^ ions emission at 567 nm; (B) RT emission spectra excited at Ce^3+^ ions at 440 nm; (C) 2D excitation–emission matrix for NDY-11% crystal; (D) Temperature dependence of integrated Ce^3+^ ions emission recorded in the 470–750 nm spectral range; (E) RT photoluminescence decay curves of Ce^3+^ emission at 567 nm excited at 440 nm; (F) Temperature-dependent evolution of the effective decay time of Ce^3+^ ions emission at 567 nm in SC, NDY-3%, NDY-6%, NDY-9%, and NDY-11% crystals. Uncertainties of τ_eff_, obtained by error propagation through eq 2, are smaller than the symbol size.

### 3.5 Photoconversion Properties

Figure 5 compares the photoconversion spectra of the SC, NDY-3%, NDY-6%, NDY-9%, and NDY-11% crystals under 450 nm laser-diode excitation (Figure 5A) and 455 nm LED excitation (Figure 5B), together with the corresponding CIE 1931 chromaticity coordinates (Figure 5C) and the derived correlated color temperature (CCT), color rendering index (CRI), and luminous efficacy (LE) values (Table 3).^63^ The emission consists of the excitation line superimposed on a broad Ce^3+^ band extending from 500 to 750 nm. The excitation source is much narrower for the laser diode (fwhm ∼ 4.3 nm) than for the LED (fwhm ∼23.7 nm). As a result, composition-dependent changes in the balance between residual blue excitation and Ce^3+^ down-converted emission are directly reflected in the chromaticity coordinates and in the corresponding CCT, CRI, and LE values.

**Figure 5:**
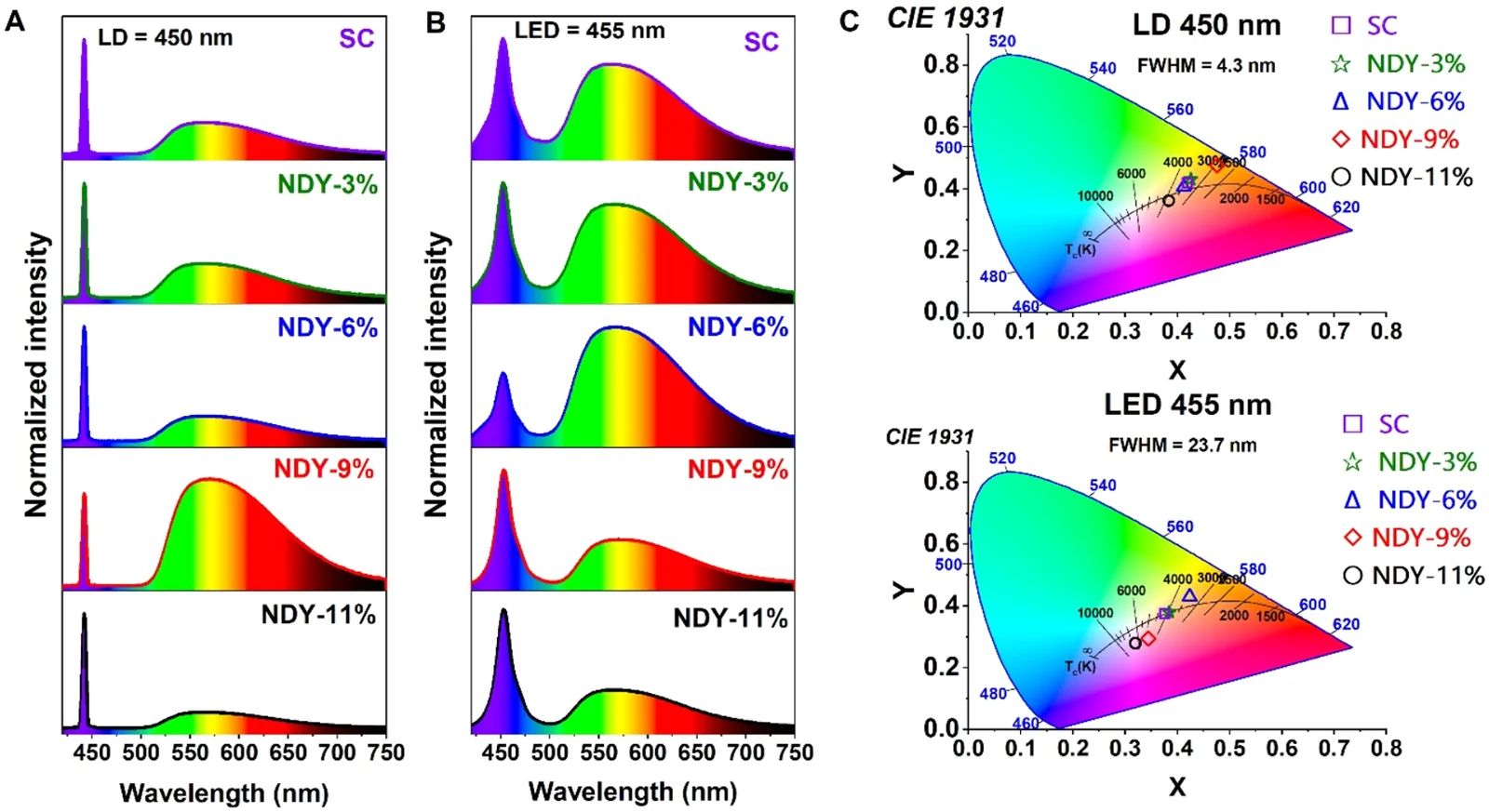
Photoconversion emission spectra of SC, NDY-3%, NDY-6%, NDY-9%, and NDY-11% crystals under (A) 450 nm laser-diode (LD) excitation and (B) 455 nm light-emitting-diode (LED) excitation. (C) CIE 1931 (x,y) chromaticity coordinates calculated from the corresponding emission spectra and plotted on the CIE 1931 diagram for LD (upper panel) and LED (lower panel) excitations.

**Table 3:** Calculated CCT, CRI and LE Parameters for the Photoconversion Spectra of SC, NDY-3%, NDY-6%, NDY-9%, and NDY-11% Crystals^a^

	**LD**			**LED**		
**sample ID**	CCT (K)	CRI	LE (lm/W)	CCT (K)	CRI	LE (lm/W)
SC	3428	57	135	4107	69	143
NDY-3%	3400	58	95	3908	68	148
NDY-6%	3471	59	113	3437	63	147
NDY-9%	2941	55	130	4716	76	158
NDY-11%	3779	59	113	6482	80	141

a The uncertainties are ±50 K for CCT, ±2 for CRI, and ±5% relative for LE, based on the calibration of the spectroradiometer.

Because CCT and CRI are determined by the spectral power distribution, whereas LE is a photopic-weighted quantity that favors emission near the maximum of V­(λ), these metrics provide a compact description of the balance between transmitted blue excitation and Ce^3+^ down-converted emission. Table 3 shows a consistent excitation-source effect across the entire series: LED excitation yields higher CRI and higher LE than LD excitation for all compositions (LED: CRI = 63–80, LE = 141–158 lm/W; LD: CRI = 55–59, LE = 95–135 lm/W). This trend is consistent with the different excitation conditions of LEDs and laser diodes, including the narrower line width and higher radiance of LD pumping, which can alter the balance between converted and unconverted light and thereby shift the derived colorimetric metrics.^64^ Within each excitation mode, the composition dependence is distinctly nonmonotonic. Under LD excitation, NDY-9% gives the warmest emission (CCT = 2941 K), consistent with a relatively stronger contribution from the Ce^3+^ conversion band, whereas NDY-11% shifts toward cooler white (CCT = 3779 K), indicating a larger residual blue fraction. Under LED excitation, NDY-11% produces the coolest white (CCT = 6482 K) and the highest CRI (80), while NDY-9% combines high color quality with the highest LE (CCT = 4716 K, CRI = 76, LE = 158 lm/W), indicating a favorable redistribution of spectral power toward the green-yellow region without a pronounced loss of spectral breadth. By contrast, NDY-6% yields the warmest white light (CCT = 3437 K) but the lowest CRI (63), consistent with a more strongly bimodal spectrum dominated by residual blue excitation and a single broad conversion band. The nonmonotonic composition dependence is consistent with the Y_2_O_3_-deficiency microstructure of the crystals, in which NDY-6% and NDY-9% contain α-Al_2_O_3_ inclusions, whereas NDY-11% contains a perovskite secondary phase. These different secondary-phase constitutions, together with their associated inclusion-matrix interfaces, may influence pump scattering, optical path length, residual blue transmission, and local heating under excitation, and thus contribute to the observed shifts in chromaticity, CCT, CRI, and LE.

### 3.6 Scintillation Properties

Figure 6A compares the temperature-dependent radioluminescence spectra of the SC, NDY-9%, and NDY-11% crystals measured between 10 and 350 K, whereas the corresponding data for NDY-3% and NDY-6% are shown in Figure S8. Across the full temperature range, the RL spectra are dominated by Ce^3+^ emission from the garnet phase and do not exhibit additional UV bands that could be assigned to defect-related luminescence.^2,4,65^ Likewise, the NDY-11% crystal shows no distinct RL features attributable to the perovskite secondary phase, despite the presence of perovskite inclusions identified by the structural analyses.^2,4,22^ This behavior indicates that scintillation under X-ray excitation is governed primarily by Ce^3+^ emission from the garnet matrix. The absence of resolved defect- or perovskite-related bands is consistent with the high intensity of the garnet Ce^3+^ emission and with efficient Tb→Ce energy transfer, which together can mask weaker contributions from minor luminescent centers or secondary phases.

**Figure 6:**
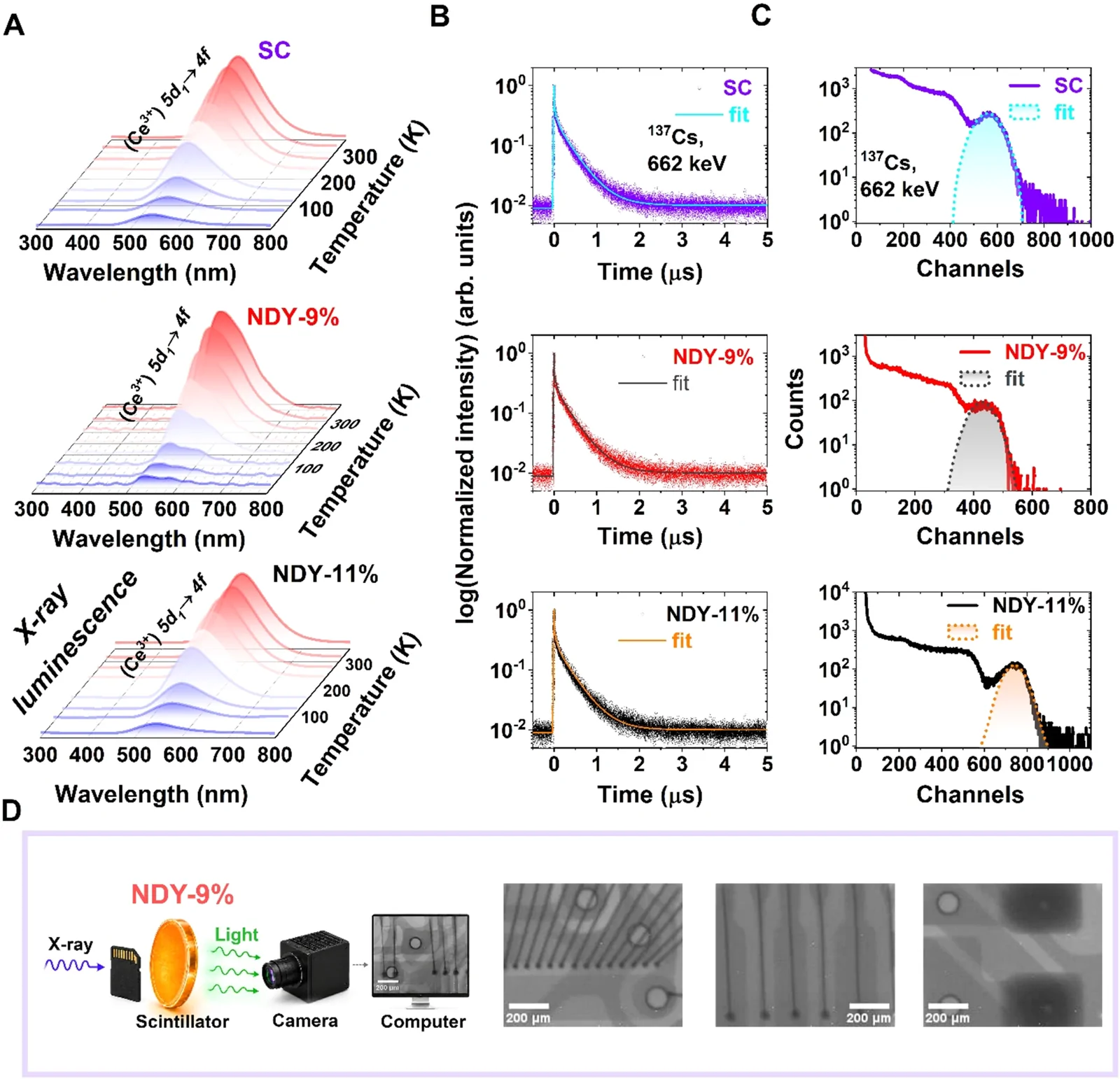
(A) Temperature-dependent radioluminescence of SC, NDY-9% and NDY-11% crystals; (B) Scintillation decay times, and (C) pulse height spectra of γ-rays from ^137^Cs radioisotope, measured for SC, NDY-9% and NDY-11% crystals, (D) schematic of the μ-X-ray imaging setup using the NDY-9% crystal as the scintillator, with representative X-ray radiographs of an SD card.

The Ce^3+^ ions associated with the perovskite phase may also contribute to the Ce^3+^ emission from the garnet phase. In particular, previous studies have suggested that excitation captured by Ce^3+^ in the perovskite phase can be transferred to the Tb^3+^ sublattice and subsequently transferred to Ce^3+^ centers in the garnet phase, thereby providing an additional sensitization pathway in NDY-11%.^4,66^ In this framework, Ce_(perovskite phase)_^3+^ *→* Tb_(perovskite/garnet sublattices)_^3+^ *→* Ce_(garnet phase)_^3+^ transfer sequence could contribute to the enhanced excitation efficiency of the NDY-11% crystal.^66^ The increase in Ce^3+^ emission intensity at elevated temperature can be explained by two complementary effects. First, shallow trapping centers become thermally depopulated as the available thermal energy exceeds their activation barriers. These trapping states may be associated with *Y*_*Al*_^*x*^ antisite defects and trace impurities including Mn^2+^, Cr^3+^, Yb^3+^.^67^ Their thermal emptying reduces parasitic carrier trapping and thereby increases the fraction of excitation energy available for radiative recombination at Ce^3+^ centers.^47,68^ Second, higher temperature can enhance phonon-assisted energy transfer from the Tb^3+^ sublattice to Ce^3+^, which provides an additional channel for populating the Ce^3+^ excited state.^62^ Figure 6B shows the scintillation decay times for SC, NDY-9% and NDY-11% crystals after excitation with γ radiation from the ^137^Cs radioisotope.

Table 4 summarizes the scintillation decay times and light-yield values. The decay curves were fitted with a multiexponential function according to eq 1, and an effective decay time (τ_eff_) was calculated using eq 2 to enable quantitative comparison of the scintillation dynamics. Relative to the single-phase crystal (1980 ns), all Y_2_O_3_-deficient crystals exhibit longer effective decay times (2500–4380 ns), consistent with the trends observed in the photoluminescence decay kinetics. The light yield varies nonmonotonically across the series and can be divided into three regimes corresponding to the structural changes described in Section 3.1. First, the largest increase occurs already between SC and NDY-3% (21,300 → 32,100 ph/MeV, ∼51%), i.e., before any secondary phase is detectable by PXRD or Raman spectroscopy. This LY enhancement may correlate with the pronounced Tb enrichment of the garnet phase in the Y_2_O_3_-deficient crystals (Table 2, Figure S2), which increases the density of Tb–Ce interaction pairs and thus the efficiency with which the Tb^3+^ sublattice transfers the ionization energy to the Ce^3+^ emission centers. Second, over the 3–9 mol % Y_2_O_3_ deficiency range the light yield is nearly constant (32,100, 32,000, and 33,600 ph/MeV), consistent with the saturation of Tb enrichment in the garnet phase (Tb ≈ 2.6–2.8 in NDY-9%, Table 2). The modest additional gain in NDY-9% may reflect improved light extraction through scattering at α-Al_2_O_3_ inclusions and defect-rich interfacial regions.^69^ Third, at 11 mol % deficiency the light yield decreases to 30,700 ph/MeV, coinciding with perovskite formation and the return of the garnet-phase Tb/Y ratio toward SC-like values (Table 2), which reduces the density of Tb–Ce pairs and weakens Tb-mediated sensitization. The fact that the light yield nevertheless remains ∼44% higher than that of SC is consistent with the additional Ce^3+^ (perovskite) → Tb^3+^ → Ce^3+^ (garnet) sensitization and with the lower concentration of deep traps in this crystal (Section 3.3).^66^ This indicates that rare-earth partitioning is the primary mechanism by which melt nonstoichiometry controls scintillation output, with secondary-phase light scattering providing a supplementary, extraction-related contribution. The maximum light yield (NDY-9%) is ∼58% higher than that of SC and ∼87% higher than that of the Ce^3+^-doped Y_3_Al_5_O_12_ reference measured under identical conditions. The role of light transport in the measured light yield is corroborated by the room-temperature transmittance spectra (Figure S9). The SC, NDY-3%, NDY-6%, and NDY-9% crystals are transparent across the Ce^3+^ emission range, with the transmittance above 520 nm increasing from approximately ∼52–54% (SC, NDY-3%) to ∼60% (NDY-6%) and ∼71% (NDY-9%). The comparatively high transparency of the α-Al_2_O_3_-bearing crystals is consistent with the confinement of the inclusions to the rim region (Section 3.1), which leaves the central volume of the crystal optically clear. The highest light yield of the series thus coincides with the lowest internal optical losses. In contrast, NDY-11% exhibits a weakly wavelength-dependent transmittance of only ∼4–5% (Figure S9B), indicative of strong light scattering at the perovskite core and the garnet-perovskite phase boundaries. Its nevertheless high light yield (30 700 ph/MeV) is consistent with the measurement geometry, in which the Teflon reflector returns multiply scattered photons to the photodetector, while the scattering itself suppresses photon trapping by total internal reflection.^69^ Notably, no defect-related absorption bands are resolved in the visible range for any composition (Figure S9), indicating that the composition-dependent differences in scintillation output arise from energy-transfer, charge-trapping, and light-transport effects.

**Table 4:** Scintillation Decay Time, Light Yield Values Measured at 10 μs Shaping Time and Energy Resolution for Single-phase Garnet and Secondary Phase-Bearing Garnet Crystals as Well as for the Ce^3+^ -Doped Y_3_Al_5_O_12_ Reference

	**decay times** (ns)/**weight** (%)					
**sample ID**	**τ**_**1**_ **(w**_**1**_ **)/error**	**τ**_**2**_ **(w**_**2**_ **)/error**	**τ**_**3**_ **(w**_**3**_ **)/error**	**τ**_**eff**_	**light yield** (photons/MeV)/**error**	**energy resolution** (%)/**error**
SC	38 (8)/ ± 4	260 (11)/ ± 13	2400 (81)/ ± 72	1980/ ± 70	21,300/ ± 1000	19.7/ ± 0.9
NDY-3%	37 (5)/ ± 3	750 (6)/ ± 29	2860 (89)/ ± 86	2500/ ± 82	32,100 ± 1600	23.8/ ± 1.5
NDY-6%	34 (4)/ ± 2	540 (6)/ ± 27	3700 (90)/ ± 110	3400/ ± 97	32,000 ± 1600	18.3/ ± 1.1
NDY-9%	45 (3)/ ± 4	560 (7)/ ± 28	4700 (90)/ ± 141	4380/ ± 122	33,600 ± 1700	16.8/ ± 0.85
NDY-11%	56 (4)/ ± 5	330 (8)/ ± 15	3060 (88)/ ± 90	2780/ ± 97	30,700 ± 1500	10.2/ ± 0.45
Y_3_Al_5_O_12_:Ce - reference	70 (39)/ ± 6	810 (61)/ ± 41	n/a	520/ ± 18	18,000 ± 900	6.8/ ± 0.34

The energy resolution of the 662 keV full-absorption peaks, obtained from Gaussian fits of the pulse-height spectra (Figure 6C), is listed in Table 4. The stoichiometric SC crystal exhibits an energy resolution equal to 19.7%, whereas the nonstoichiometric crystals show 23.8, 18.3, 16.8, and 10.2% for NDY-3%, NDY-6%, NDY-9%, and NDY-11%, respectively, compared with 6.8% for the Ce^3+^-doped YAG reference. Across the series, energy resolution does not follow the trend in light yield. NDY-3% exhibits the poorest energy resolution despite a light yield ∼51% higher than that of SC, and NDY-11% achieves the best resolution although its light yield is lower than that of NDY-9%. The observed differences in energy resolution are attributed primarily to inhomogeneity of the scintillation response, arising from the radial Tb/Y and Ce concentration gradients (Figure 1D, Table 2) and from the spatial distribution of the secondary phases, which make the light output position-dependent and broaden the photopeak.^70^ Radial EPMA profiles show that all crystals exhibit pronounced segregation of the activator, with Ce enriched near the rim and depleted in the central region (Figure 1D for SC, NDY-9%, and NDY-11%; Figure S2 for NDY-3% and NDY-6%), along with radial variations of the Tb/Y ratio on the rare-earth sublattice. Such compositional gradients make the local scintillation efficiency position-dependent and broaden the full-absorption peak, and the spatial distribution of the secondary phases introduces a further source of position-dependent light collection. The improvement observed from NDY-3% to NDY-11% is consistent with the corresponding microstructural changes across the series. The α-Al_2_O_3_ inclusions in NDY-6% and NDY-9% are confined to the rim region, whereas in NDY-11% the secondary phase is restricted to the perovskite core, and the surrounding garnet matrix regains a radially uniform, SC-like Tb/Y composition (Table 2). The lower concentration of deep traps in NDY-11% (Section 3.3) may further reduce fluctuations in the collected light by limiting trap-mediated delayed recombination.^69^

Quantitative analysis of the TL glow curves in the 30–550 K temperature range (Figure 3A,B and S4A and S4B; Tables S1 and S2) shows that the magnitude of trap-related contributions does not scale directly with the increase in scintillation light yield. Instead, the light-yield enhancement correlates more closely with the pronounced increase in τ_eff_, as summarized in Figure 6B and Table 4. This comparison suggests that the improvement in scintillation performance is governed primarily by changes in excited-state dynamics and not by trap populations alone. Increasing Y_2_O_3_ deficiency drives the garnet lattice away from ideal stoichiometry, promotes secondary-phase formation, and modifies the local environment of Tb^3+^ and Ce^3+^ ions in the garnet phase. These changes are expected to alter Tb↔Ce energy-transfer rate and the competition between radiative recombination and trapping.

To evaluate the practical relevance of the enhanced scintillation response, X-ray imaging was performed using the NDY-9% crystal as the scintillating converter. This composition was selected because it exhibits the highest scintillation light yield in the series, reaching 33 600 photons/MeV under ^137^Cs γ-ray excitation (Table 4). Figure 6D presents a schematic of the imaging configuration together with representative radiographic images of an SD card. In this setup, transmitted X-rays are converted by the NDY-9% crystal into visible Ce^3+^ emission, which is optically collected by the camera and reconstructed as a contrast image. The resulting radiographs clearly resolve the metallic contacts and internal microstructural features of the SD card, demonstrating the practical X-ray imaging capability of the high light yield α-Al_2_O_3_-bearing NDY-9% crystal. These results show that controlled Y_2_O_3_ deficiency is an effective approach for improving scintillation output and achieving application-relevant radiographic performance in Ce^3+^-doped mixed garnet crystals.

### 3.7 Electron Paramagnetic Resonance Spectra

The EPR spectra recorded before X-ray irradiation are essentially identical for all crystals; therefore, only the spectrum of the perovskite-bearing NDY-11% crystal is shown in Figure 7A. In all cases, the spectra are dominated by a single broad resonance band. At 70 K, only its high-field side is resolved within the accessible magnetic-field range. Upon cooling from 50 to 30 K, the resonance shifts to higher magnetic field and increases in intensity. On further cooling to 20 K, however, the line narrows markedly, its intensity decreases, and the resonance shifts slightly back toward lower field. This nonmonotonic evolution of resonance position, line width, and intensity is consistent with the development of spin–spin correlations on cooling and with changes in exchange relaxation in the vicinity of a magnetic transition. Accordingly, the simultaneous line narrowing and loss of intensity at 20 K suggest the onset of magnetic ordering.^71^

**Figure 7:**
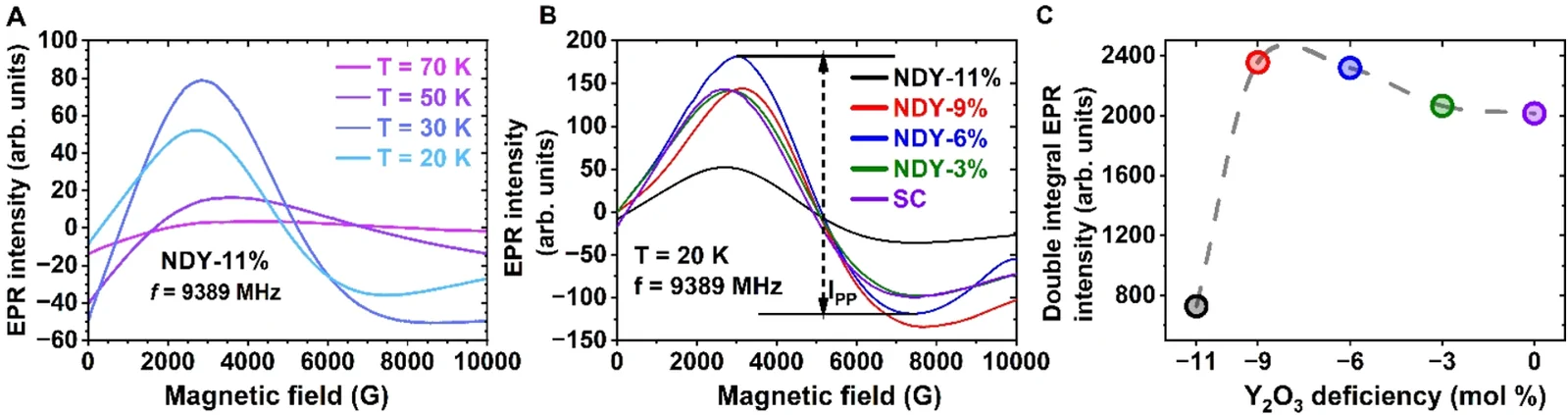
(A) X-band EPR spectra of the perovskite-bearing NDY-11% crystal measured at different temperatures before X-ray irradiation; (B) X-band EPR spectra of SC, NDY-3%, NDY-6%, NDY-9% and NDY-11% crystals measured at *T* = 20 K, I_pp_ stands for the peak-to-peak intensity; (C) Double integral EPR intensity plotted as a function of Y deficiency concentration.

The exceptionally large line width, approaching 3500 G at 20 K (Figure 7A), together with the high Tb concentration of these crystals, is consistent with assignment of the broad EPR signal to Tb-related paramagnetic centers, most likely Tb^3+^ ions.^72^ Such strong broadening is expected in Tb-rich garnets because dipolar and exchange interactions become substantial at high rare-earth concentrations. This assignment is also chemically reasonable for an aluminum-garnet lattice dominated by Tb^3+^ ions. No resolved Ce^3+^ resonance is observed. Given the large population of paramagnetic Tb centers and the measurable Ce content, Tb–Ce exchange interactions are expected and may contribute to the absence of a distinct Ce^3+^ signal.^73^ The same strong exchange coupling can also explain why no resolved irradiation-induced EPR features are detected after X-ray exposure in either the single-phase SC crystal or multiphase crystals. Any paramagnetic trapping centers generated during irradiation are likely to remain strongly coupled to the dominant Tb sublattice and therefore unresolved within the broad Tb resonance. A similar absence of resolvable features has been demonstrated in prior work on the Eu^2+^-F^+^ system (F+ appears when oxygen vacancy traps an electron).^74^ To examine how Y_2_O_3_- deficiency affects Tb incorporation into the garnet host, EPR spectra were collected for the full compositional series from 0 to −11 mol % (Figure 7B). Both the line width and the peak-to-peak signal intensity change systematically with composition. For quantitative comparison, the double integral (DI) of the EPR signal was evaluated (Figure 7C). Under identical measurement conditions, DI is proportional to the concentration of paramagnetic centers.^73^ The DI reaches a maximum for NDY-9% and is nearly three times larger than for NDY-11%, whereas only a modest decrease is observed between NDY-6% and SC. These trends indicate that Y_2_O_3_-deficiency strongly affects the concentration of Tb-related paramagnetic centers, in agreement with the Tb content determined independently from the compositional analysis. The markedly lower DI for NDY-11% is consistent with redistribution of Tb during formation of the perovskite secondary phase, which reduces the concentration of Tb-related paramagnetic centers in the garnet matrix. More generally, the nonmonotonic DI dependence on the Y_2_O_3_ deficiency shows that Y deficiency modifies the local magnetic environment of Tb. Charge-compensation effects, possible changes in Tb valence (Tb^3+^↔Tb^4+^) arising from local charge imbalance relative to the stoichiometric garnet, and variation in Tb–Tb and Tb–Ce exchange and dipolar interactions may all contribute. However, the strong line broadening prevents reliable separation of individual species. Accordingly, the EPR results are best interpreted in terms of composition-dependent changes in the population and local environment of Tb-related paramagnetic centers.

## 4 Conclusions

This work demonstrated that controlled melt nonstoichiometry governed both the type and spatial distribution of secondary phases in Ce^3+^-doped (Tb,Y)_3_Al_5_O_12_ crystals grown by the micropulling-down method. The progression from phase-pure garnet through rim-localized α-Al_2_O_3_ to core-localized perovskite was determined by the melt chemistry. Since the Tb_2_O_3_ concentration was held constant, increasing Y_2_O_3_ deficiency did not deplete the melt of rare-earth cations but reduced the availability of Y^3+^ ions, a key component for stabilizing the congruently melting garnet phase. As the system approached the binary Tb_2_O_3_–Al_2_O_3_ system, in which the garnet melts incongruently and TbAlO_3_ is the stable primary phase, phase competition shifted from garnet-corundum to garnet-perovskite. This phase competition was accompanied by a distinct spatial separation across the crystal cross-section, with the garnet-Al_2_O_3_ eutectic confined to the rim and the perovskite confined to the core. The secondary phases influenced the functional properties of the crystals by altering the Tb/Y ratio of the surrounding garnet matrix during their formation. The composition of the rare-earth sublattice governed the bidirectional Tb^3+^↔Ce^3+^ energy exchange and consequently affected the delayed component of the Ce^3+^ emission, its thermal stability, and the scintillation response. Comparison of the thermoluminescence trap parameters with the scintillation output showed that the enhanced light-yield in the multiphase crystals originated primarily from excited-state dynamics and light extraction. The microstructure played a dual role, as inclusion-matrix interfaces introduced deep trapping centers while the same inhomogeneities improved photon out-coupling. The α-Al_2_O_3_-bearing crystal grown at 9 mol % Y_2_O_3_ deficiency exhibited the highest luminous efficacy and scintillation light yield, with sufficient performance for X-ray radiography of a microelectronic device, whereas the perovskite-bearing crystal at 11 mol % deficiency provided the highest thermal stability of the Ce^3+^ emission (T_90_ increasing from 375 to 425 K) and the best color rendering performance. Melt nonstoichiometry therefore enabled a balance between white-light conversion and radiation-detection performance within single garnet system, without changing the dopant, host cation set, or growth technique. This phase-engineering approach may be applied to other rare-earth aluminate systems in which one end-member garnet melts incongruently. All crystals were grown at a fixed pulling rate of 0.05 mm/min to isolate the effect of melt nonstoichiometry on phase selection. The melt composition determined which secondary phase formed, since the phase constitution was governed by melt phase equilibria, whereas the solidification rate influenced the scale and radial distribution of that phase and the effective segregation coefficients of the constituent cations. A systematic study of the pulling-rate dependence would clarify the extent to which the microstructure and associated photoconversion and scintillation properties can be varied at a fixed melt composition.

## Supplementary Material



## Notes

Powder XRD patterns and Rietveld refinements of secondary-phase fractions in NDY-9% and NDY-11% crystals; SEM-EDS elemental maps and EPMA radial atomic-ratio profiles of Tb, Y, Al, and Ce; spatially resolved Raman spectra from rim to center; thermoluminescence glow curves with peak deconvolution, time-dependent fading, and partial thermal-cleaning (*T*_max_–*T*_stop_) analysis; fitted shallow- and deep-trap parameters; temperature-dependent photoluminescence excitation and emission spectra and Ce3+ decay curves; temperature-dependent radioluminescence, pulse-height spectra, and scintillation decay profiles; and room-temperature optical transmittance spectra (PDF)
