# Supplementary material for: Yttrium Oxide-Deficient Melts Control Secondary-Phase Distribution in Cerium-Doped Terbium–Yttrium Aluminum Garnet Crystals for White-Light Conversion and X‑ray Imaging
Source: Chem Mater. 2026 Aug 26;38(17):8987–9003. doi: 10.1021/acs.chemmater.6c01589 (PMC13560952; doi:10.1021/acs.chemmater.6c01589)
Supplement: Supplementary file 1 [file cm6c01589_si_001.pdf]

Yttrium Oxide-Deficient Melts Control Secondary-Phase Distribution in Cerium-Doped Terbium-Yttrium Aluminum Garnet Crystals for White-Light Conversion and X-ray Imaging

Karol Bartosiewicz[1]*, Justyna Zeler[2], Marcin E. Witkowski[3], Masao Yoshino[4], Robert Tomala[5], Om Prakash[6], Damian Szymanski[5], Maksym Buryi[7], Aleksandra Owczarek[2], Andrey Prokhorov[1,7], Vítězslav Jarý[1], Kei Kamada[4,8], Pietro Galinetto[6], Winicjusz Drozdowski[3], Eugeniusz Zych[2], Akira Yoshikawa[4,8], Gongxun Bai[9]

[1] Institute of Physics of the Czech Academy of Sciences, Na Slovance 1999/2, 182 00 Prague 8, Czechia
[2] Faculty of Chemistry, University of Wrocław, Joliot-Curie 14F, 50-383 Wrocław, Poland
[3] Institute of Physics, Faculty of Physics, Astronomy and Informatics, Nicolaus Copernicus University in Toruń, Grudziądzka 5, 87-100 Toruń, Poland
[4] New Industry Creation Hatchery Center, Tohoku University, 6-6-10 Aramaki Aza Aoba, Aoba-ku, Sendai, Miyagi 980-8579, Japan
[5] Institute of Low Temperature and Structure Research, Polish Academy of Sciences, Okólna 2, 50-422 Wrocław, Poland
[6] Department of Physics, University of Pavia, Via Bassi 6, 27100 Pavia, Italy
[7] Institute of Plasma Physics of the Czech Academy of Sciences, U Slovanky 2525/1a, 182 00 Prague 8, Czechia
[8] Institute for Materials Research, Tohoku University, 2-1-1 Katahira, Aoba-ku, Sendai, Miyagi 980-8577, Japan
[9] College of Science, China Jiliang University, Hangzhou 310018, China

* Email: bartosiewicz@fzu.cz

Supporting Information

1. *PXRD, Rietveld, SEM-EDS, and EPMA analysis of Y_2O_3 deficiency effect on phase stability and atom segregation*

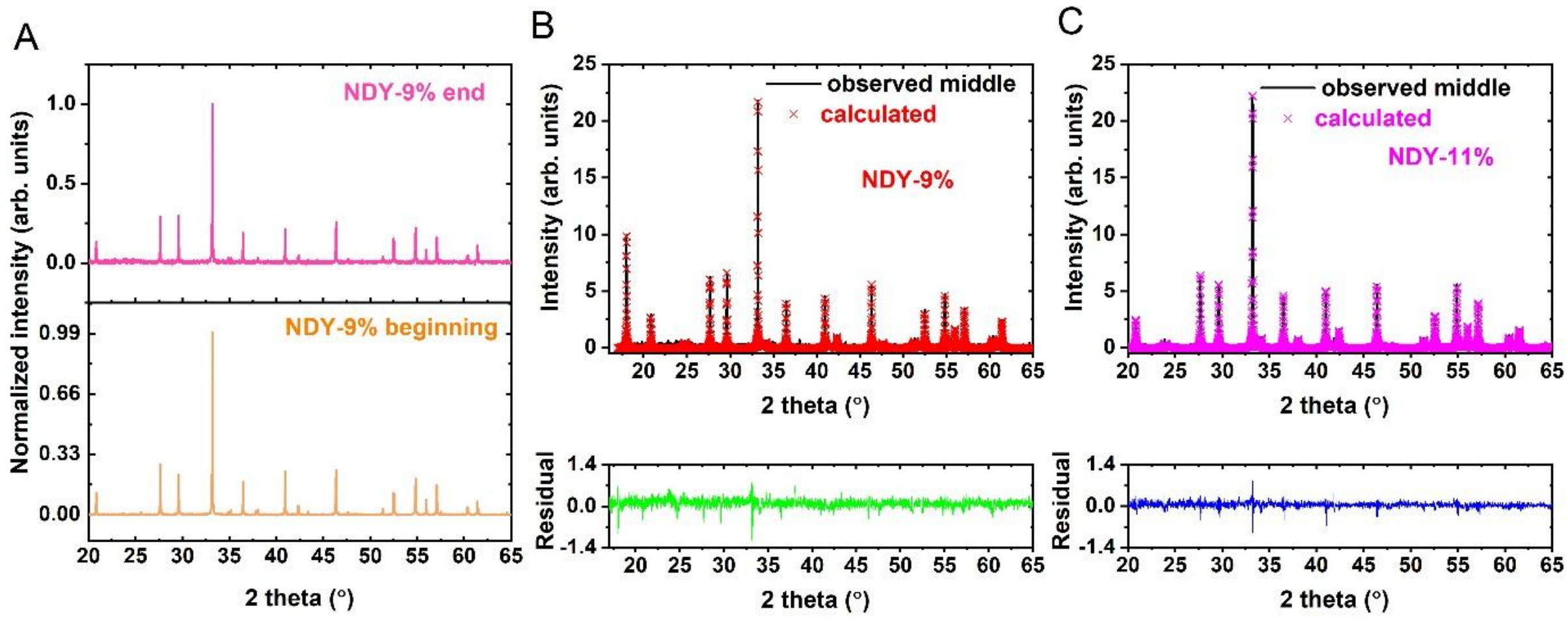

Figure S1. (A) PXRD patterns of NDY-9% recorded at the beginning and the end of the crystal rod. Rietveld refinement for (B) NDY-9% and (C) NDY-11% crystals.

Figure S1A presents a comparison of the powder X-ray diffraction patterns obtained from the initial and terminal segments of the NDY-9% crystal rod. While the overall phase composition remains consistent, a slight variation is observed in the intensity of the diffraction peaks associated with the α-Al_2O_3 phase. Specifically, a reduced intensity of these peaks is noted in the section extracted from the terminal portion of the crystal rod, suggesting a slightly lower concentration of the α-Al_2O_3 phase in this region. This reduction may be attributed to a volume-related effect arising from the geometry of the crystal rod, which narrows towards a conical tip. The reduced cross-sectional area in this conical zone could limit the incorporation of the α-Al_2O_3 phase during solidification, likely due to spatial constraints and altered growth dynamics in the narrowing region.

Structural refinement was performed using the Rietveld refrainment, with the *Jana2020* software to quantitatively analyze the phase composition of the multi-phase crystals, as illustrated in Figures S1B and S1C.[1] The refinement was used to estimate the volume fractions of the garnet and α-Al_2O_3 phases in the NDY-9% crystal and the garnet and perovskite phases in the NDY-11% crystal. In both compositions, the garnet phase was found to be dominant. For the NDY-9% crystal, the α-Al_2O_3 phase accounted for approximately 4.78 vol% (± 0.5%), while in the NDY-11% crystal, the perovskite phase constituted 3.91 vol% (± 0.5%) of the total

structure. These results confirm that minor secondary phases are present, yet garnet remains the primary structural component across both hypoeutectic compositions.

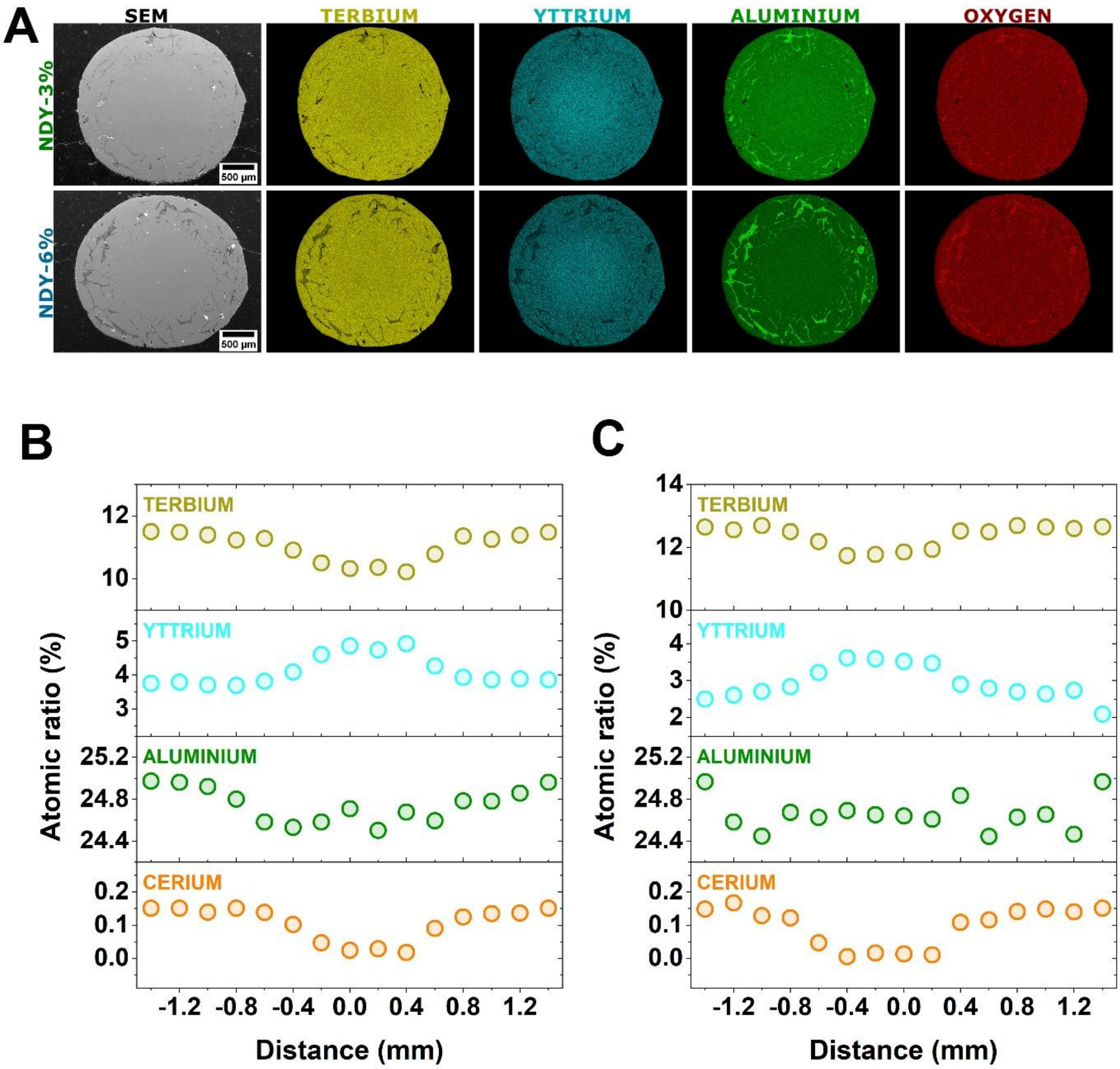

Figure S2. (A) EDS elemental maps of terbium (yellow), yttrium (cyan), aluminum (green), and oxygen (red), along with EPMA radial atomic ratio profiles of Tb, Y, Al and Ce for the Al_2O_3-bearing garnet phases (B) NDY-3% and (C) NDY-6% crystals.

The EDS elemental maps for terbium, yttrium, aluminum, and oxygen in the NDY-3% and NDY-6% samples are shown in Figure S2A. In both samples, distinct regions near the crystal rims exhibit enhanced signals for aluminum and oxygen, accompanied by comparatively weaker signals for terbium and yttrium. A similar trend is observed in the local elemental fluctuations measured via electron probe microanalysis, further supporting this spatial variation (Figure S2B and S2C). These local enrichments in Al and O suggest the possible presence of

Al_2O_3-rich inclusions. However, no secondary phases were detected by X-ray diffraction (Figure 1 in main article), indicating that, if present, the concentration of such phases is likely below the detection limit of the XRD technique, which is typically estimated at around 2% by volume.[2]

2. Raman spectroscopy

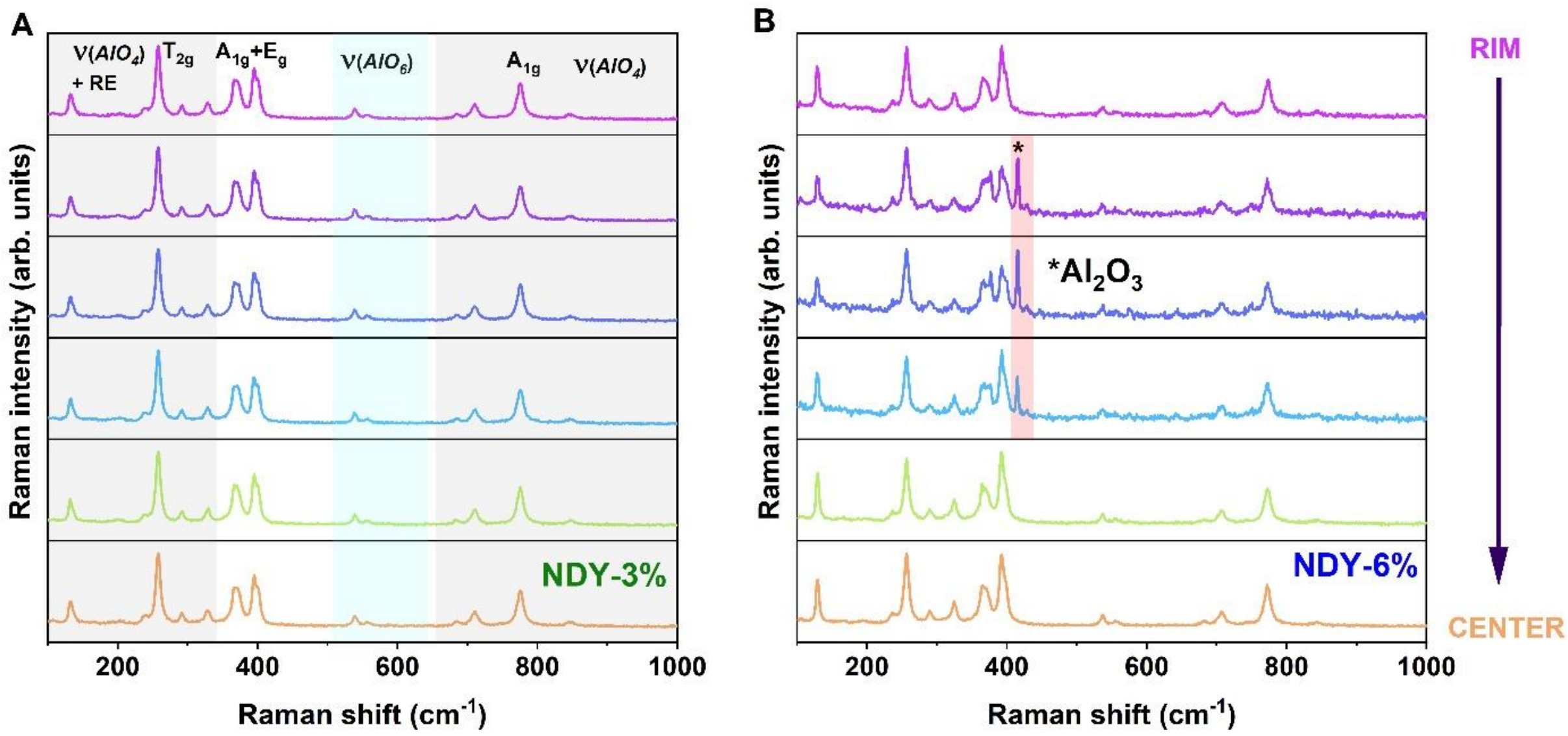

Figure S3. Raman spectra collected along a linear scan from the rim to the center of the (A) NDY-3% and (B) NDY-6% samples.

Relative to NDY-9%, NDY-3% exhibits several additional low-frequency Raman bands (~150 - 250 cm^{-1}), an increased relative intensity of the AlO_4 stretching modes (~800 - 850 cm^{-1}), and a slight red shift of selected bands above 600 cm^{-1}. These spectral changes are consistent with the higher Tb content in NDY-3%, which increases the average cation mass and perturbs the local lattice environment. The additional low-frequency features likely reflect Tb-related lattice vibrations and coupled cation motions activated by local symmetry reduction, whereas the altered balance between stretching and bending modes suggests modification of the local Al–O coordination environment by lattice distortion and strain. The Raman spectra of NDY-6% are intermediate between those of NDY-3% and NDY-9%, with weak shoulder bands at 300 - 350 cm^{-1}, stretching-mode intensities similar to those of NDY-3%, and band positions closer to those of NDY-9%, indicating partial lattice distortion and the coexistence of Tb- and Y-related vibrational contributions. In addition, spatially resolved Raman spectra reveal an

additional sharp band at ~418 cm^{-1} in the rim and rim-center regions of the NDY-6% sample, identical to the feature observed for NDY-9%. This band is consistent with the presence of α-Al_2O_3 and therefore indicates that NDY-6% also contains α-Al_2O_3 inclusions. This interpretation is supported by the SEM-EDS and EPMA results, whereas the absence of a corresponding PXRD reflection suggests that the α-Al_2O_3 fraction remains below the detection limit of the PXRD method (approximately 2%).

3. Thermoluminescence analysis

The TL glow curve analysis presented in Figures S4A-C provides insight into the trap characteristics in NDY-3% and NDY-6% crystals. Figure S4A and S4B show the TL glow curves deconvoluted into individual trap components across low (30 - 300 K) and high (300 - 550 K) temperature ranges. The TL glow curves for NDY-3% and NDY-6% show a multi-peak structure at low temperature (30 - 300 K), indicating several shallow traps that empty at relatively low thermal activation, together with a dominant high-temperature band in the 480–520 K region (300 - 550 K) that signifies a substantial deep-trap population.[3] The time-dependent TL glow curves for NDY-3% and NDY-6% (Figure S4C) show the same qualitative fading pattern. The lower-temperature contributions decrease strongly with storage time, while the high-temperature band near 480 - 520 K remains comparatively stable and is still detectable after long delays. Consistent with their α-Al_2O_3 secondary phase, the persistence of the 480 - 520 K band supports a significant deep-trap population, plausibly associated with defect-rich garnet-Al_2O_3 interfaces and vacancy-related centers.[3, 4] To better understand the trapping centers in studied crystals, partial thermal cleaning measurements were performed (Figure S4D and S4E). In this method, the samples ware irradiated with the same X-ray dose, preheated to a selected T_{stop}, cooled to room temperature, and then measured by standard TL readout. As T_{stop} increased from 50 to 250 °C, shallow traps were progressively emptied and deeper, more stable traps became dominant. At the highest T_{stop} values, the TL signal became nearly negligible. Between about 50 and 95 °C, the low-temperature peak decreased, while the relative contributions of peaks near 130 and 220 °C increased. This may suggest thermally induced redistribution or retrapping into deeper traps, although overlap between glow-curve components could also contribute. The T_{max}–T_{stop} plot shows three nearly flat regions, indicating groups of closely related traps with narrow trap-depth distributions rather than one broad continuum.

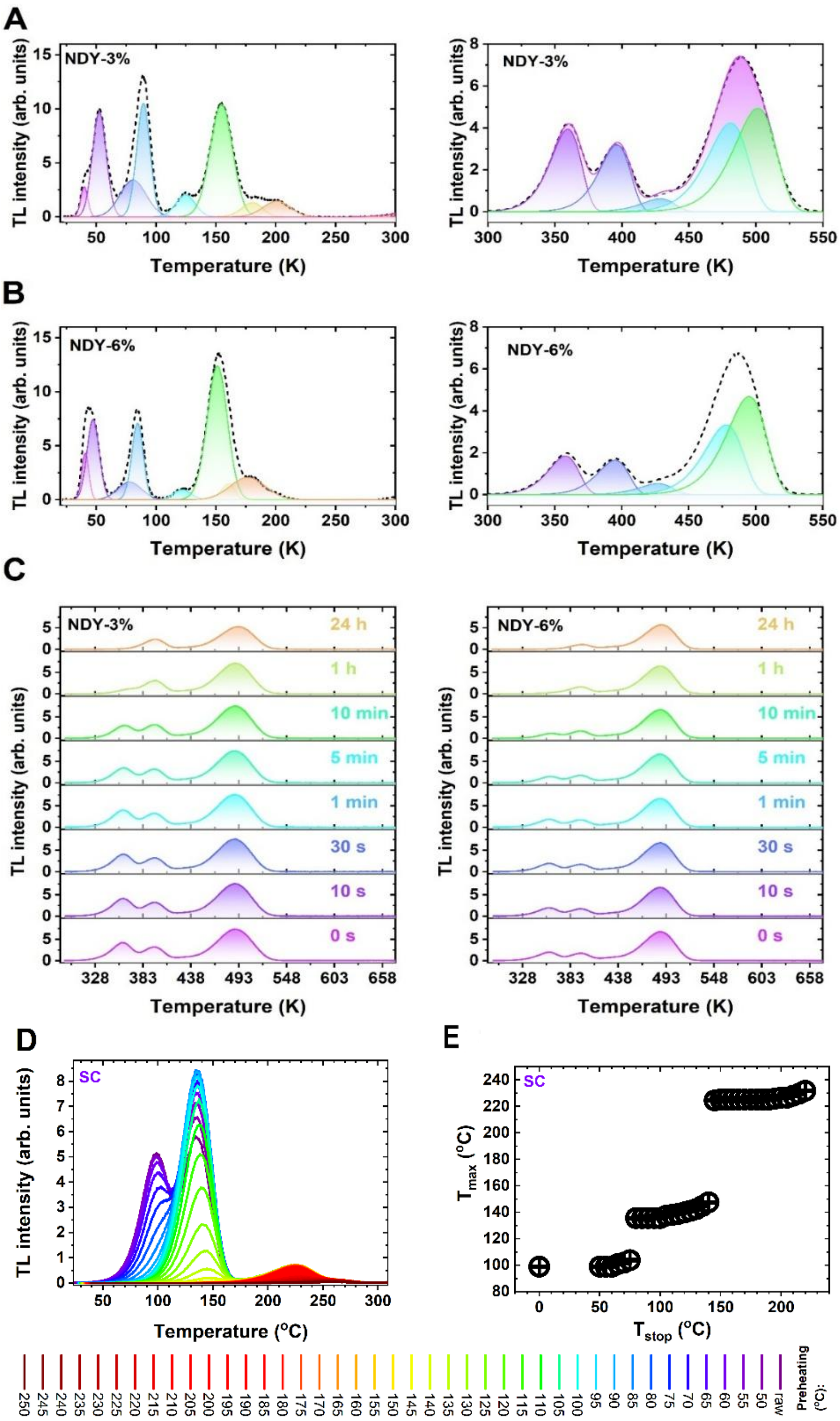

Figure S4. Thermoluminescence glow curves of (A) NDY-3% and (B) NDY-6% crystals recorded after X-ray irradiation between 30-550 K; (C) time-dependent thermoluminescence curves between 0 s and 24 h after irradiation for NDY-3% and NDY-6% crystals. Each glow curve was deconvoluted into individual peaks to resolve individual trap contributions. (D) TL glow curves recorded after X-ray irradiation and preheating to different T_{stop} values (50-250 °C), followed by cooling and standard TL measurement. Increasing T_{stop} removes low-temperature components, narrows the curves, and shifts T_{max} to higher temperatures. (E) T_{max}-T_{stop} dependence showing three step-like regions, indicating three main groups of closely related traps.

Table S1. The shallow trap parameters detected in SC and NDY-3%, NDY-6%, NDY-9%, NDY-11% crystals obtained from fitting include the peak temperature (T_{max}), activation energy (E), and amplitude (A). The uncertainties resulting from the fitting procedure are ±3 K for T_{max}, ±5% relative for E, and ±5% relative for A.

Sample ID	Parameters	TL peak number							
		1	2	3	4	5	6	7	8
SC	T_{max} (K)	50	60	90	95	130	170	200	n/a
	E (meV)	140	169	268	277	378	480	573	n/a
	A ($x10^5$)	3.59	4.97	17.4	5.36	7.69	38.3	8.3	n/a
NDY-3%	T_{max} (K)	40	53	80	89	124	154	180	201
	E (meV)	114	151	235	260	366	457	536	598
	A ($x10^5$)	2.09	142	9.36	147	4.16	2.45	2.93	4.18
NDY-6%	T_{max} (K)	40	47	78	84	122	151	160	177
	E (meV)	116	136	226	245	358	472	447	525
	A ($x10^5$)	3.22	9.52	4.14	8.53	1.73	2.16	2.64	7.67
NDY-9%	T_{max} (K)	40	42	74	78	113	134	146	174
	E (meV)	114	124	217	228	332	432	428	514
	A ($x10^5$)	2.86	1.02	1.29	2.95	0.65	4.44	4.16	2.54
NDY-11%	T_{max} (K)	45	52	80	92	140	185	215	n/a
	E (meV)	123	141	232	267	408	497	620	n/a
	A ($x10^5$)	1.02	1.86	5.88	1.26	7.43	2.41	1.71	n/a

Table S2. The deep trap parameters detected in SC and NDY-3%, NDY-6%, NDY-9%, NDY-11% crystals obtained from fitting include the peak temperature (T_{max}), activation energy (E), and frequency factor (s). The uncertainties resulting from the fitting procedure are ±3 K for T_{max} and ±0.05 eV for E, and the frequency factors are reliable within a factor of three due to the correlation between E and s in the fit.

Sample ID	Parameters	TL peak number					
		1	2	3	4	5	6
SC	T_{max} (K)	362	372	401	414	483	506
	E (eV)	0.97	1.12	1.21	1.23	1.31	1.45
	s ($x10^{14}$, s^{-1})	0.13	6.9	6.7	3.6	15.0	0.86
NDY-3%	T_{max} (K)	360	n/a	396	428	481	501
	E (eV)	0.96	n/a	1.16	1.27	1.30	1.35
	s ($x10^{14}$, s^{-1})	12.3	n/a	2.44	3.49	13.6	0.116
NDY-6%	T_{max} (K)	358	n/a	395	427	478	495
	E (eV)	0.95	n/a	1.14	1.25	1.35	1.40
	s ($x10^{14}$, s^{-1})	10.2	n/a	1.49	2.26	58.8	59.5
NDY-9%	T_{max} (K)	351	n/a	388	427	470	488
	E (eV)	0.94	n/a	1.10	1.25	1.28	1.32
	s ($x10^{14}$, s^{-1})	13.9	n/a	82.4	2.26	17.9	13.8
NDY-11%	T_{max} (K)	363	372	402	418	490	506
	E (eV)	1.01	1.07	1.21	1.25	1.32	1.38
	s ($x10^{14}$, s^{-1})	1.0	2.8	13.0	9.7	0.27	0.37

4. Temperature dependence of photoluminescence emission and excitation spectra

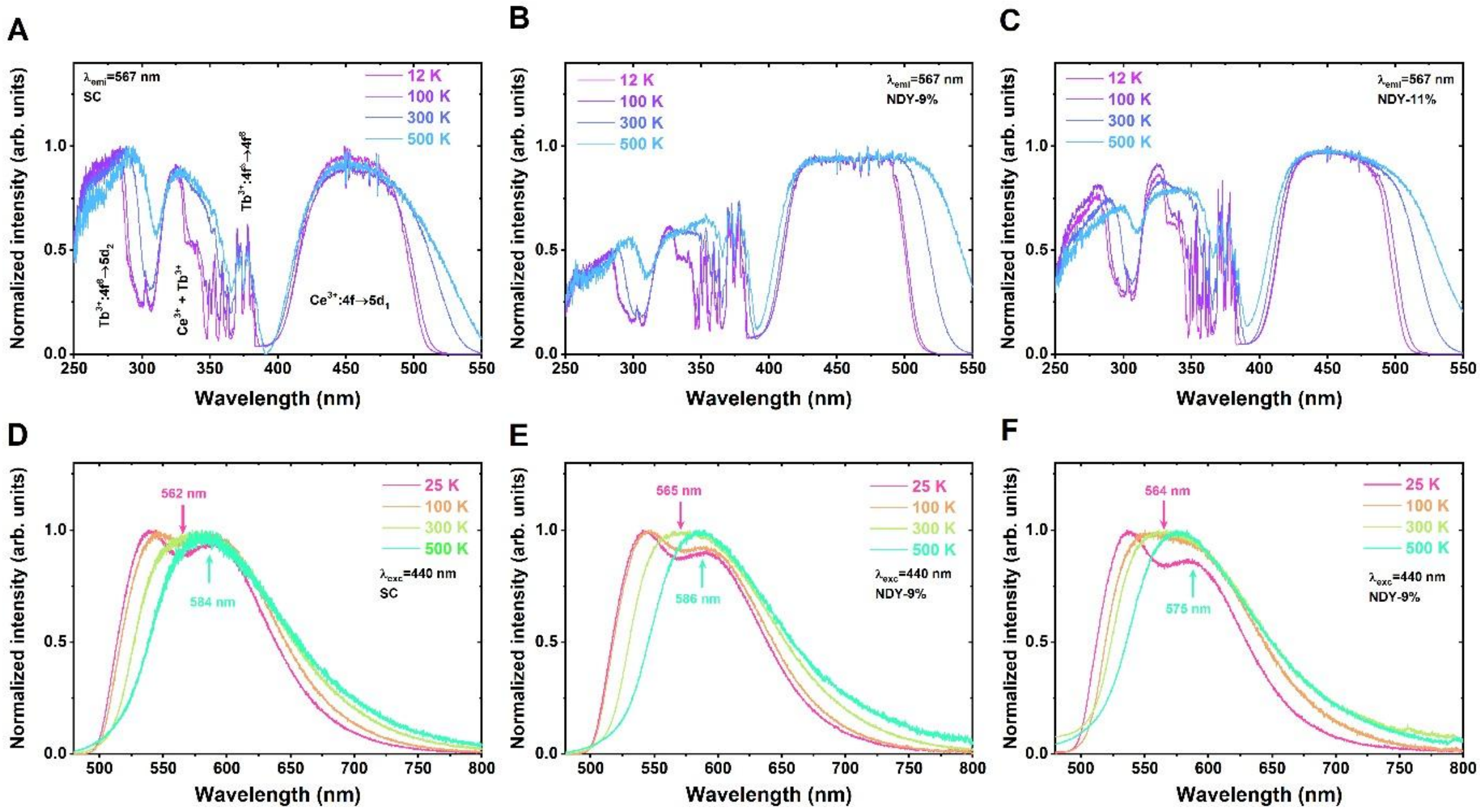

Figure S5. Temperature dependence of the photoluminescence (A-C) excitation spectra (λ_{emi} = 567 nm) and (D-F) emission spectra (λ_{exc} = 440 nm) of the SC, NDY-9% and NDY-11% crystals recorded between 12 and 500 K.

Figure S5 shows the photoluminescence-excitation spectra for the Ce^{3+} ions emission at 567 nm and the photoluminescence-emission spectra excited at 440 nm for SC, NDY-9% and NDY-11% crystals recorded in the 12 - 650 K temperature range. In all samples the excitation and emission bands gradually broaden with increasing temperature, leading to the increase of their spectral overlap, and consequently, to a significant enhancement of energy-transfer processes within the Ce^{3+}-Ce^{3+} pairs.

Figure S6A, S6B and S6C presents the temperature-dependent behavior of overlapping Ce^{3+} and Tb^{3+} emissions under excitation at 270 nm into the $4f \rightarrow 5d_1$ energy level of Tb^{3+} ions. At low temperatures (<50 K) and very high temperatures (>550 K), Tb^{3+} emission intensity is dominant in SC single phase and NDY-11%. At lower temperatures, the $^5D_4$ (Tb^{3+}) and $5d_1$ (Ce^{3+}) energy states are not in close resonance, hindering energy transfer between Tb^{3+} and Ce^{3+} ions, resulting in the dominance of Tb^{3+} luminescence. With increasing temperature, the Ce^{3+} emission becomes more prominent due to the phonon-mediated energy transfer process from Tb^{3+} to Ce^{3+} facilitated by the host lattice.

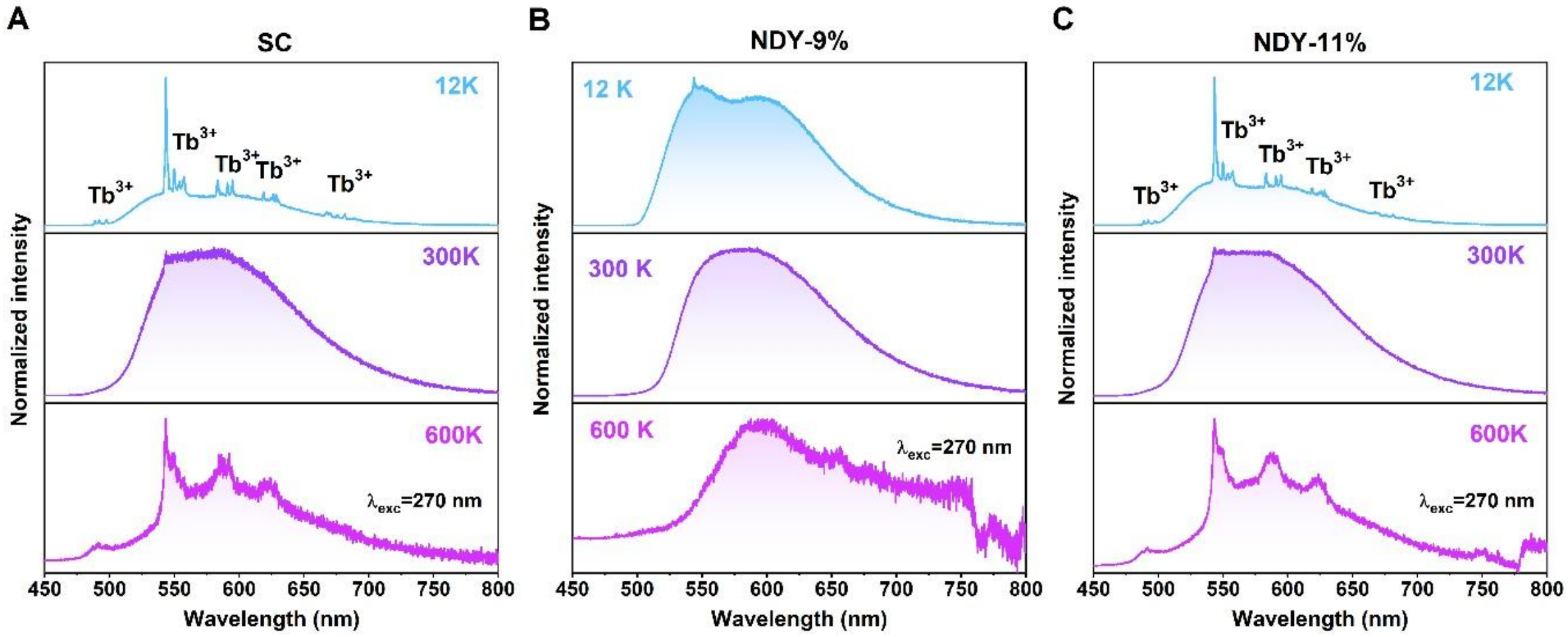

Figure S6. The photoluminescence emission spectra for (A) SC, (B) NDY-9% and (C) NDY-11% crystals excited at 270 nm into the $5d_1$ energy state of Tb^{3+} ions measured as a function of temperature from 12 to 650 K.

At temperatures above 550 K, the Ce^{3+} emission is significantly quenched due to thermal depopulation of the $5d_1$ state, causing Tb^{3+} emission intensity to regain its dominance. A markedly different temperature dependence of the $Tb^{3+}\rightarrow Ce^{3+}$ energy transfer process is observed in the NDY-9% hypoeutectic structure. In this sample, Ce^{3+} emission remains dominant across the entire temperature range, from low temperatures (12 K) to elevated temperatures (650 K), with only weak and barely discernible emission lines from Tb^{3+} ions. This behavior indicates a highly efficient energy transfer from Tb^{3+} to Ce^{3+} ions, likely due to the optimal resonance between the $^5D_4$ energy state of Tb^{3+} and the $5d_1$ state of Ce^{3+} ions. The energy levels are sufficiently well-aligned to allow direct transfer without the assistance of host lattice phonons to bridge an energy mismatch. A similar finding has been reported in Ce^{3+}-doped $Tb_3Al_{5-x}Sc_xO_{12}$ crystals, which also exhibit a multiphase microstructure.[5]

Figure S7 presents the selected decay curves of Ce^{3+} ions emission at 567 nm, excited at 440 nm (Ce^{3+}, $4f\rightarrow 5d_1$ transition), for SC and NDY-11% crystals, recorded at temperatures of 12, 300, and 500 K. The decay curves were analyzed using a multiexponential fitting function as described by Equation 1. At 12 K, the decay times for both crystals exhibit a single exponential character with decay times of 59 ns, which is typical for isolated Ce^{3+} ions in garnets.[6] At 300 K and 500 K, the decay times display a multiexponential character, comprising three distinct components. This multiexponential behavior reflects the back-and-forth Ce^{3+}-to-Tb^{3+} energy transfer processes.[7] In the NDY-11% crystal, the decay constants are slightly longer compared

to those in SC sample. This difference can be attributed to enhanced reabsorption in the NDY-11% crystal, due to the presence of a secondary phase which acts as a light-scattering center. [8]. Additionally, the higher structural disorder in NDY-11% crystal results in better resonance between the Tb^{3+} and Ce^{3+} energy levels, thereby elongating the back-and-forth Ce^{3+}-to-Tb^{3+} energy transfer process.[9]

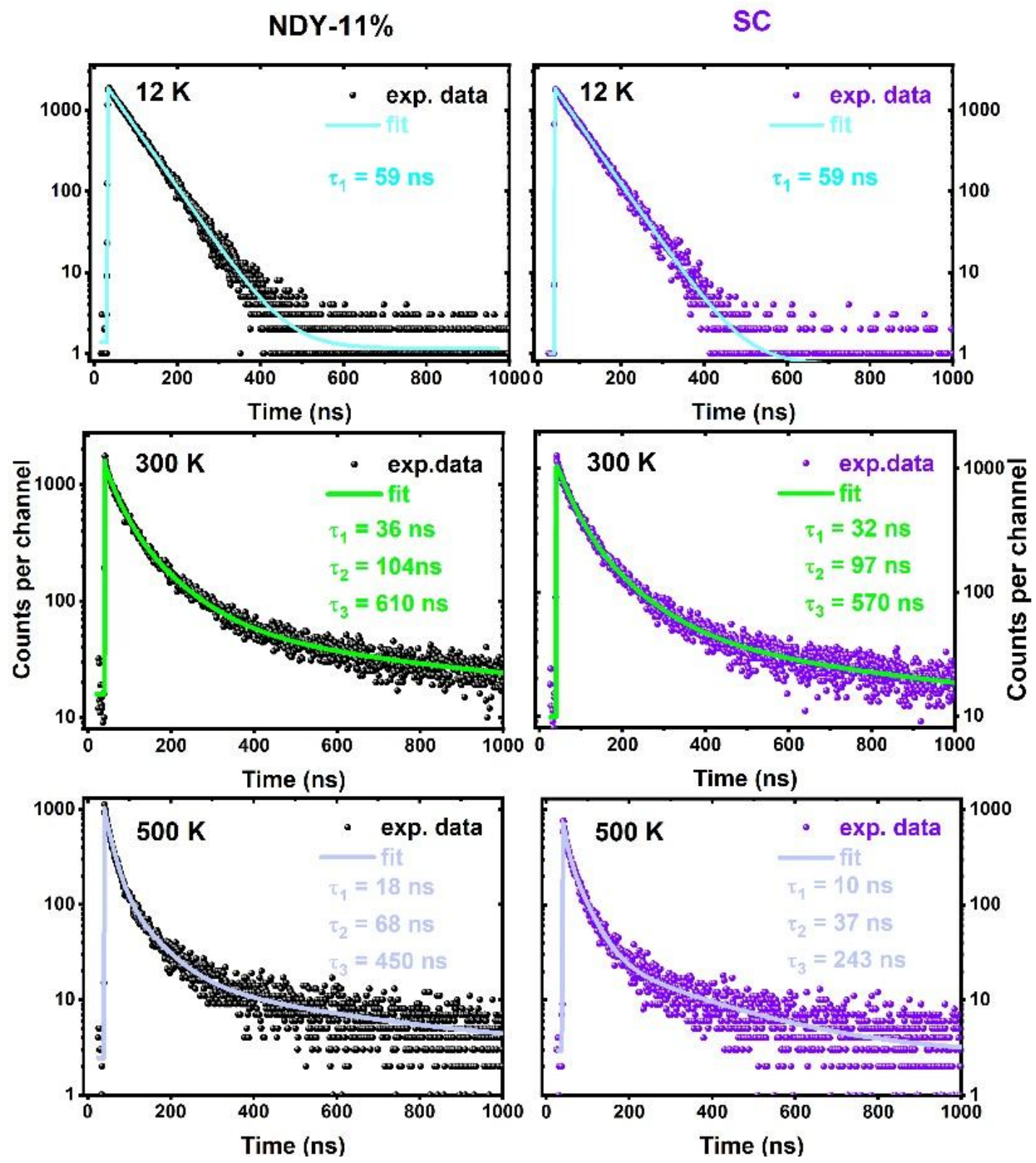

Figure S7. Selected decay curves of Ce^{3+} emission at 567 nm, excited at 440 nm (Ce^{3+} $4f \rightarrow 5d_1$ transition) for NDY-11% and SC crystals, recorded at temperatures of 12, 300, and 500 K.

5. *Scintillation properties*

Figure S8 shows scintillation properties of the NDY-3% and NDY-6% crystals. Figure S8A displays radioluminescence spectra recorded between 12 and 300 K, dominated by the characteristic broad Ce^{3+} ions emission band in the visible range, over this interval, the spectral profile remains largely unchanged, while the intensity and peak position exhibit a clear temperature dependence. Figure S8B presents the corresponding pulse-height spectra. Figure S8C shows the scintillation decay profiles measured over the microsecond time window.

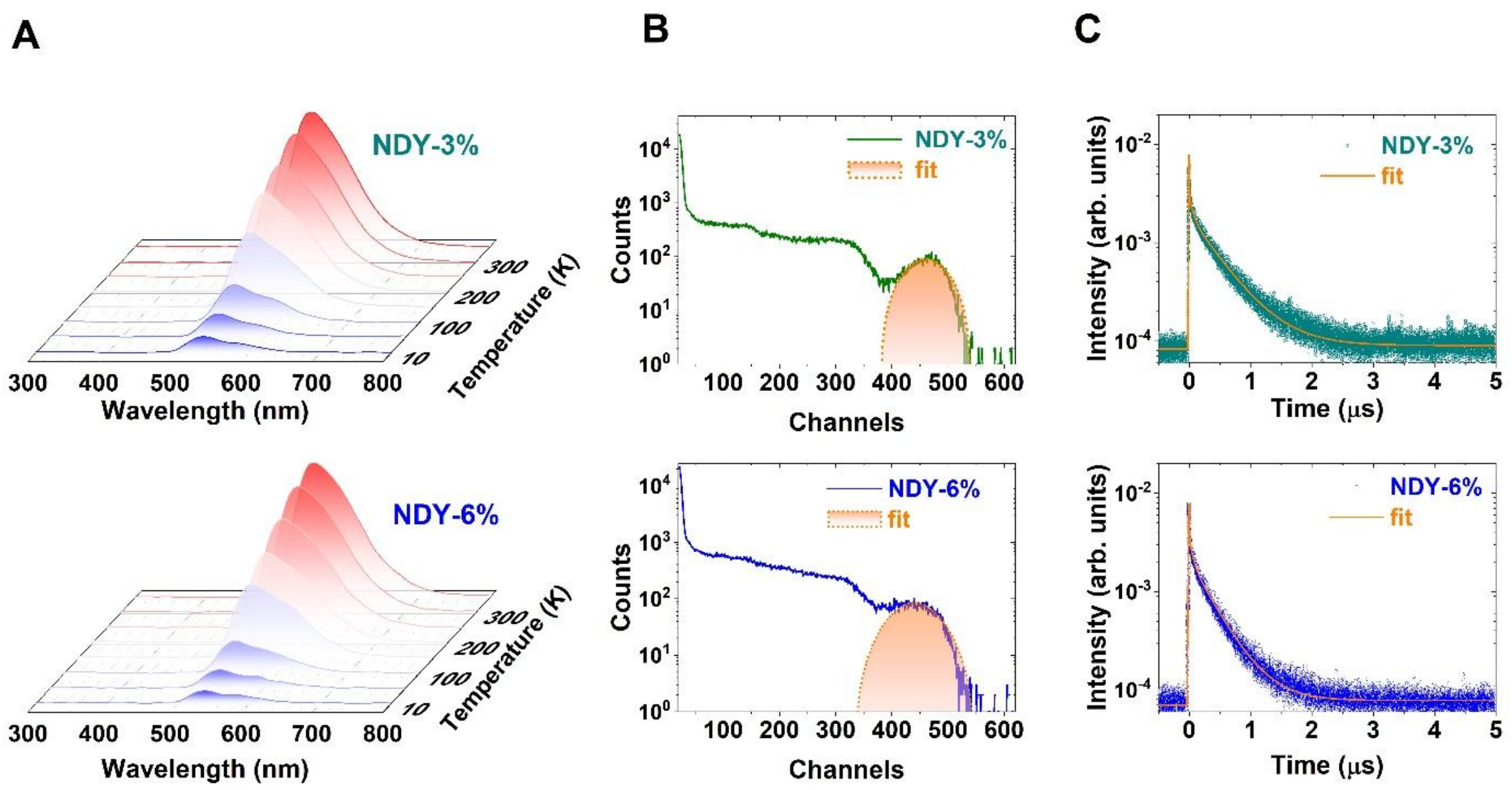

Figure S8. (A) Temperature-dependent x-ray exited luminescence of nonstoichiometric NDY-3% and NDY-6% crystals; (B) pulse height spectra and (C) scintillation decay times of γ-rays from ^{137}Cs radioisotope, measured for nonstoichiometric NDY-3% and NDY-6% crystals.

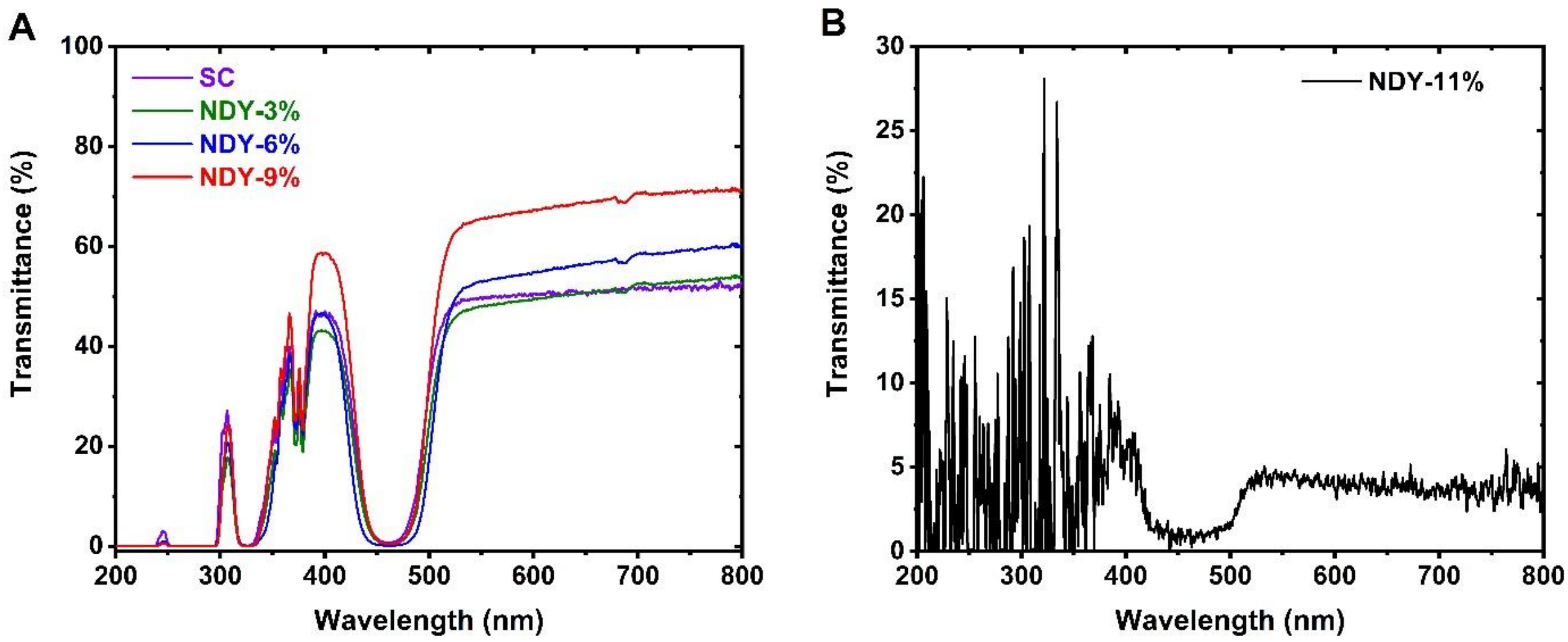

Figure S9. Room-temperature optical transmittance spectra measured in the 200-800 nm spectral range for (A) the SC, NDY-3%, NDY-6%, and NDY-9% crystals and (B) the NDY-11% crystal. Note the different transmittance scale in panel (B).

Figure S9 presents the room-temperature transmittance spectra of all crystals. The SC, NDY-3%, NDY-6%, and NDY-9% crystals (Figure S9A) exhibit a common absorption structure consistent with the excitation spectra in Figure 4A. This structure comprises complete absorption below ~290 nm associated with the spin-allowed *4f* → *5d* transitions of Tb^{3+} (~220 and 275 nm), a Ce^{3+} $4f \rightarrow 5d_2$ absorption band at ~325-345 nm, sharp Tb^{3+} *4f–4f* absorption

lines between ~340 and 385 nm, and a fully saturated Ce^{3+} *4f → 5d₁* band at ~440-500 nm. Above ~520 nm, i.e., across the Ce^{3+} emission range, the crystals are transparent, with transmittance increasing from ~52-54% (SC, NDY-3%) to ~60% (NDY-6%) and ~71% (NDY-9%). The comparatively high transparency of the α-Al_2O_3-bearing crystals is consistent with the confinement of the inclusions to the rim region (Section 3.1), which leaves the central volume of the crystal comparatively free of scattering inclusions. In contrast, the NDY-11% crystal (Figure S9B) shows a strongly reduced, weakly wavelength-dependent transmittance of only ~4-5% above 520 nm. The nearly flat spectral dependence indicates that the attenuation is dominated by light scattering at the perovskite inclusions and garnet-perovskite phase boundaries in the crystal core. No absorption bands other than those of Ce^{3+} and Tb^{3+} ions are resolved in any of the crystals.

Acknowledgments

This project has received funding from the European Union's Horizon Europe research and innovation programme under the Marie Skłodowska-Curie Actions COFUND, Physics for Future, grant agreement No. 101081515. This work was also supported by the GIMRT program of the Institute for Materials Research, Tohoku University, Proposal No. 202412-RDKYA-0503. Part of the thermoluminescence results was supported by the National Science Centre, Poland, under project No. 2023/49/B/ST5/04265. J.Z. and A.O. gratefully acknowledge this support.

References:

(1) Rietveld, H. M. A Profile Refinement Method for Nuclear and Magnetic Structures. *J Appl Crystallogr* **1969**, *2* (2), 65-71. DOI: 10.1107/S0021889869006558.
(2) Bartosiewicz, K.; Babin, V.; Kamada, K.; Yoshikawa, A.; Kurosawa, S.; Beitlerova, A.; Kucerkova, R.; Nikl, M.; Zorenko, Y. Ga for Al substitution effects on the garnet phase stability and luminescence properties of $Gd_3Ga_xAl_{5-x}O_{12}$:Ce single crystals. *J Lumin* **2019**, *216*. DOI: 10.1016/j.jlumin.2019.116724. Bartosiewicz, K.; Babin, V.; Kamada, K.; Yoshikawa, A.; Mares, J. A.; Beitlerova, A.; Nikl, M. Luminescence quenching and scintillation response in the Ce^{3+} doped $Gd_xY_{3-x}Al_5O_{12}$ (x = 0.75, 1, 1.25, 1.5, 1.75, 2) single crystals. *Opt Mater* **2017**, *63*, 134-142. DOI: 10.1016/j.optmat.2016.05.041.
(3) Bos, A. J. J. Thermoluminescence as a Research Tool to Investigate Luminescence Mechanisms. *Materials* **2017**, *10* (12). DOI: 10.3390/ma10121357.
(4) Uberuaga, B. P.; Dholabhai, P. P.; Pilania, G.; Chen, A. P. Semicoherent oxide heterointerfaces: Structure, properties, and implications. *Apl Materials* **2019**, *7* (10). DOI: 10.1063/1.5121027.
(5) Bartosiewicz, K.; Tomala, R.; Szymanski, D.; Albini, B.; Zeler, J.; Yoshino, M.; Horiai, T.; Socha, P.; Kurosawa, S.; Kamada, K.; et al. Micro-Inclusion Engineering via Sc Incompatibility for Luminescence

and Photoconversion Control in Ce^{3+}-Doped $Tb_3Al_{5-x}Sc_xO_{12}$ Garnet. *Materials* **2024**, *17* (11). DOI: 10.3390/ma17112762.
(6) Bartosiewicz, K.; Szysiak, A.; Tomala, R.; Gołębiewski, P.; Węglarz, H.; Nagirnyi, V.; Kirm, M.; Romet, I.; Buryi, M.; Jary, V.; et al. Energy-Transfer Processes in Nonstoichiometric and Stoichiometric Er^{3+}, Ho^{3+}, Nd^{3+}, Pr^{3+}, and Cr^{3+}-Codoped Ce:YAG Transparent Ceramics: Toward High-Power and Warm-White Laser Diodes and LEDs. *Physical Review Applied* **2023**, *20* (1). DOI: 10.1103/PhysRevApplied.20.014047.
(7) Bartosiewicz, K.; Babin, V.; Kamada, K.; Yoshikawa, A.; Nikl, M. Energy migration processes in undoped and Ce-doped multicomponent garnet single crystal scintillators. *J Lumin* **2015**, *166*, 117-122. DOI: 10.1016/j.jlumin.2015.05.015.
(8) Lagorio, M. G.; San Román, E. How Does Light Scattering Affect Luminescence? Fluorescence Spectra and Quantum Yields in the Solid Phase. *Journal of Chemical Education* **2002**, *79* (11). DOI: 10.1021/ed079p1362. Tomasini, E. P.; Román, E. S.; Braslavsky, S. E. Validation of Fluorescence Quantum Yields for Light-Scattering Powdered Samples by Laser-Induced Optoacoustic Spectroscopy. **April 17, 2009**. DOI: 10.1021/la803492. Martini, M.; Montagna, M.; Ou, M.; Tillement, O.; Roux, S.; Perriat, P. How to measure quantum yields in scattering media: Application to the quantum yield measurement of fluorescein molecules encapsulated in sub-100 nm silica particles. *Journal of Applied Physics* **2009/11/01**, *106* (9). DOI: 10.1063/1.3248302.
(9) Bartosiewicz, K.; Babin, V.; Nikl, M.; Mares, J. A.; Zorenko, Y.; Gorbenko, V. Luminescence and energy transfer processes in $(Lu,Tb)_3Al_5O_{12}$ single crystalline films doped with Ce^{3+}. *J Lumin* **2016**, *173*, 141-148. DOI: 10.1016/j.jlumin.2016.01.022.
